# Cooperative nuclear action of RNA‐binding proteins PSF and G3BP2 to sustain neuronal cell viability is decreased in aging and dementia

**DOI:** 10.1111/acel.14316

**Published:** 2024-08-18

**Authors:** Ken‐ichi Takayama, Takashi Suzuki, Kaoru Sato, Yuko Saito, Satoshi Inoue

**Affiliations:** ^1^ Department of Systems Aging Science and Medicine Tokyo Metropolitan Institute for Geriatrics and Gerontology Itabashi Tokyo Japan; ^2^ Department of Anatomic Pathology Tohoku University Graduate School of Medicine Sendai Miyagi Japan; ^3^ Department of Pathology Tohoku University Hospital Sendai Miyagi Japan; ^4^ Integrated Research Initiative for Living Well with Dementia Tokyo Metropolitan Institute for Geriatrics and Gerontology Tokyo Japan; ^5^ Department of Neuropathology (the Brain Bank for Aging Research) Tokyo Metropolitan Institute for Geriatrics and Gerontology Itabashi Tokyo Japan; ^6^ Division of Systems Medicine and Gene Therapy Saitama Medical University Saitama Japan

**Keywords:** aging, Alzheimer type of dementia, androgen, estrogen, paraspeckle, RNA‐binding protein, stress granule

## Abstract

Dysfunctional RNA‐binding proteins (RBPs) have been implicated in several geriatric diseases, including Alzheimer's disease (AD). However, little is known about the nuclear molecular actions and cooperative functions mediated by RBPs that affect gene regulation in sporadic AD or aging. In the present study, we investigated aging‐ and AD‐associated changes in the expression of PSF and G3BP2, which are representative RBPs associated with sex hormone activity. We determined that both PSF and G3BP2 levels were decreased in aged brains compared to young brains of mice. RNA sequencing (RNA‐seq) analysis of human neuronal cells has shown that PSF is responsible for neuron‐specific functions and sustains cell viability. In addition, we showed that PSF interacted with G3BP2 in the nucleus and stress granules (SGs) at the protein level. Moreover, PSF–mediated gene regulation at the RNA level correlated with G3BP2. Interestingly, PSF and G3BP2 target genes are associated with AD development. Mechanistically, quantitative reverse transcription‐polymerase chain reaction (qRT‐PCR) analysis demonstrated that the interaction of RBPs with the pre‐mRNA of target genes enhanced post‐transcriptional mRNA stability, suggesting a possible role for these RBPs in preserving neuronal cell viability. Notably, in the brains of patients with sporadic AD, decreased expression of PSF and G3BP2 in neurons was observed compared to non‐AD patients. Overall, our findings suggest that the cooperative action of PSF and G3BP2 in the nucleus is important for preventing aging and AD development.

## Graphical abstract

Little is known about the nuclear molecular actions and cooperative functions mediated by RNA‐binding proteins (RBPs) in sporadic Alzheimer's disease (AD) or aging. We showed reduced expression levels of PSF and G3BP2, which are representative RBPs associated with sex hormone activity, in aging and AD development. PSF interacted with G3BP2 mainly in the nucleus and regulate neuron activity associated gene expressions at the RNA level, suggesting the preventive role of both RBPs for aging‐associated diseases such as dementia.

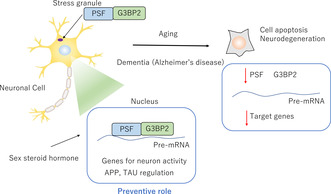

**Abbreviations**
5‐mC: 5‐methylcytosine
AD: Alzheimer's disease
APP: Amyloid Beta Precursor Protein
AR: androgen receptor
AS: sodium arsenite
CTBP1: carboxyl‐terminal binding protein 1
eIF3: elongation initiation factor 3
ER: estrogen receptor
FTD: Frontotemporal dementia
G3BP1/2: RasGAP‐associated endoribonuclease 1 and 2
H_2_O_2_
: hydrogen peroxide
HDAC: histone deacetylase
IF: Immunofluorescence
IHC: immunohistochemistry
INHBA: Inhibin Subunit Beta A
MAPT: Microtubule Associated Protein Tau
Med‐IP: methylated DNA immunoprecipitation
NEAT1: nuclear paraspeckle assembly transcript 1
NFT: neurofibrillary tangle
NONO: non‐POU domain containing octamer binding
NRG3: Neuregulin 3
PABP: poly A‐binding protein
PRKN: Parkin RBR E3 Ubiquitin Protein Ligase
PSF: polypyrimidine tract‐binding protein‐associated splicing factor
PTAU: phosphorylated TAU
qRT‐PCR: quantitative reverse transcription‐polymerase chain reaction
RBP: RNA‐binding protein
RNA‐seq: RNA sequencing
SCFD2: Sec1 family domain containing 2
SEMA5B: Semaphorin 5B
SFPQ: splicing factor, proline, and glutamine‐rich
SG: stress granule
siRNA: small interfering RNA
SIRT1: sirtuin 1
SRRM4: Serine/Arginine Repetitive Matrix 4
TIA‐1: T‐cell intracellular antigen 1
TUNEL: terminal deoxynucleotidyl transferase dUTP nick end labeling

## 1 INTRODUCTION

The effect of aging societies on the prevalence of dementia is evident, particularly in developed countries. Alzheimer's disease (AD) is the most prevalent type of dementia worldwide and is a critical threat to human health due to progressive neurodegeneration (Puri et al., 2023). Typically, a slow progression of cognitive decline characterizes sporadic AD, and a disease duration is approximately 10 years after the onset of clinical symptoms. Representative pathological features of the AD brain are extracellular Aβ plaques and intracellular neurofibrillary tangles (NFTs) of hyperphosphorylated tau protein (Lee et al., 2018; Tracy et al., 2022). Aβ pathology is supposed to trigger a cascade of additional pathological events, including the formation of NFTs, neuroinflammation, oxidative stress, and neuronal loss. The microtubule‐associated protein tau becomes hyperphosphorylated and mis‐localizes in neurons. Tau then begins to aggregate, forming large NFTs and resulting in neurodegeneration. Thus, tau deposition is a major pathological hallmark of AD (Tracy et al., 2022).

Multidomain RNA‐binding protein (RBP), polypyrimidine tract‐binding protein (PTB)‐associated splicing factor (PSF), also known as splicing factor, proline, and glutamine‐rich (SFPQ), regulate alternative splicing and transcriptional activation/repression by binding to target RNA molecules (Knott et al., 2016). PSF is predominantly a nuclear protein and a critical component of the nuclear granule called the paraspeckle (Iyer et al., 2015). Long noncoding RNA *nuclear paraspeckle assembly transcript 1* (*NEAT1*) associates largely with PSF, as well as many other RBPs, such as the non‐POU domain containing octamer binding gene (NONO), and forms paraspeckles that regulate gene expression through the nuclear retention of RNA. PSF possesses one DNA‐binding domain and two RNA recognition motifs that facilitate multiple functions in the genome and RNA processing, including epigenetics, transcription, DNA damage repair, and alternative splicing (Knott et al., 2016). PSF is an essential component of the spliceosome and is responsible for splicing pre‐mRNAs such as *APP* (Takayama et al., 2019) and *MAPT* (the transcript of tau) (Ray et al., 2011). PSF regulates the splicing of *MAPT* exon 10, which provides an additional mechanism for controlling tau aggregation by determining alternative splicing of tau transcripts. PSF target genes are essential for neuronal differentiation and development in mice (Lim et al., 2020). PSF maintains the transcriptional elongation of longer genes that are specifically expressed in neurons by binding to transcribed RNAs in the mouse brain (Takeuchi et al., 2018). Furthermore, due to a nuclear localization signal at the C‐terminus, PSF is mainly distributed in the nucleus, close to DNA damage loci and chromatin (Lim et al., 2020). PSF represses target gene transcription through epigenetic mechanism by recruiting histone deacetylase (HDAC) enzymes to specific genomic regions (Mathur et al., 2001; Takayama et al., 2013).

The sex hormones, androgen and estrogen, act by binding to their receptor proteins. The estrogen (ER) and androgen (AR) receptors are nuclear receptors that translocate to the nucleus and regulate transcription (Krause et al., 2021). Sex hormone levels are closely associated with geriatric diseases. Animal models and epidemiological studies have shown that sex hormones play a preventive role in frailty conditions such as dementia, sarcopenia, and osteoporosis (Barth et al., 2015; Krause et al., 2021). Thus, a functional decline in sex hormone signaling leads to cognitive dysfunction (Duarte‐Guterman et al., 2015). We previously proposed that PSF are associated with sex steroid hormone signaling (Mitobe et al., 2020; Takayama et al., 2013, 2017). In ERα‐positive breast cancer, PSF associates with the pre‐mRNAs of oncogenic genes, such as ERα (ESR1) and Sec1 family domain containing 2 (SCFD2), to sustain the mRNA level by post‐transcriptional regulation (Mitobe et al., 2020). We also showed that PSF binds to pre‐mRNAs of various spliceosomal factors at the RNA level and facilitates complex formation in advanced prostate cancer (Takayama et al., 2017). Dysregulation of the spliceosome complex activates the spliceosome for the production of AR and its variants through RNA‐level modifications. Moreover, an androgen‐induced lncRNA in the antisense region of *carboxyl‐terminal binding protein 1* (*CTBP1*), *CTBP1*‐*AS*, coordinates the global epigenetic status by interacting with PSF and HDAC complexes and repress cell cycle regulators or AR co‐repressors to activate AR activity (Takayama et al., 2013).

Physiological loss of PSF leads to apoptosis in the brains of mice (Takeuchi et al., 2018), suggesting a critical role for PSF in fetal brain development. Recent evidence has also shown an important role for PSF in the progression of neurodegenerative diseases (Ishigaki et al., 2017; Ke et al., 2012). The dysregulation of the PSF associated with tau proteins in AD and Frontotemporal dementia (FTD) brains has been documented (Ishigaki et al., 2017; Ke et al., 2012). PSF knockdown in the mouse brain induces FTD‐like behavior with altered tau isoform expression (Ishigaki et al., 2017). Downregulation of PSF at the protein level in AD brains has been previously observed. Braak‐stage‐dependent reductions in PSF levels have been observed in the endothelial cortex of patients with AD (Ke et al., 2012). Interestingly, the translocation of PSF to the cytoplasm has been observed in patients with rapidly progressive AD (Younas et al., 2020) and amyotrophic lateral sclerosis (ALS) (Lim et al., 2020), suggesting that PSF is important in the pathology of these diseases.

The function of RasGAP‐associated endoribonucleases 1 and 2 (G3BP1/2) depends on its N‐terminal domain, which is homologous to nuclear transporter 2 and its C‐terminal RNA recognition motif, which binds to specific RNA targets (Guillén‐Boixet et al., 2020). G3BP1 knockout in mice leads to significant akinesia, indicating its role in synaptic transmission and plasticity in the hippocampus (Martin et al., 2013). This evidence suggests an important role for G3BP1/2 in neuronal transmission. Recent advances in molecular cell biology have highlighted that G3BP1/2 play an important role in the assembly of stress granules (SGs) (Cao et al., 2020). SGs are formed by consolidation of RBPs with mRNAs via cytoplasmic translocation and translational stress switches. SGs are formed following exposure of cells to various stresses and are associated with translation inhibition. Over‐expressed G3BP1/2 induces SG formation in the absence of stress in cells, (Guillén‐Boixet et al., 2020). SGs contain other proteins such as T‐cell intracellular antigen 1 (TIA‐1), elongation initiation factor 3 (eIF3), poly A‐binding protein (PABP), and multiple RNAs (Cao et al., 2020). In a previous study, we reported that G3BP2 is a representative direct target gene of the AR, suggesting an important role for G3BP2 as a sex hormone‐related RBP (Ashikari et al., 2017). G3BP2 forms a complex with another SG‐related RBP, TRIM25, and facilitates SUMO‐mediated repression of p53 activity to induce cell cycle progression and block apoptosis. However, the pathological significance of G3BP2 expression in aging and the development of AD remains unclear.

SG‐associated RBPs play a role in tau aggregation and AD progression at the molecular level. These reports have redirected research interest toward the molecular mechanisms of RBPs in disease progression and aging (Wolozin & Ivanov, 2019). However, little is known about how RBPs are expressed in the brains of patients with AD, although it has been speculated that the gene regulatory networks controlled by RBPs influence brain function during aging. In this study, we investigated the roles of PSF and G3BP2 in aging and sporadic AD development. By analyzing protein and mRNA expression levels, we showed that PSF and G3BP2 expression levels decreased with age in mice. Epigenetic regulation, sex hormone action, and protein level regulation of PSF and G3BP2 are associated with age‐dependent decline. We then examined the gene regulatory networks mediated by PSF and G3BP2 in human neuronal cells. Global gene expression profiles of human neuronal cells revealed the essential role of PSF in maintaining neuronal cell activity. Specifically, we demonstrated that PSF interacts with G3BP2 in the nucleus to jointly regulate genes associated with neuronal activity and AD progression, indicating the importance of crosstalk between these RBP‐mediated nuclear actions in maintaining cell viability. We further analyzed brain tissues from patients with sporadic AD to investigate whether PSF and G3BP2 expression levels were modulated by AD development. We observed the nuclear and cytoplasmic localization of G3BP1 and G3BP2, as well as the nuclear enrichment of PSF. The expression levels of PSF and G3BP2 were reduced compared to controls at both protein and RNA levels, suggesting the importance of gene regulation by PSF and G3BP2 in preventing AD development in the brain. Thus, our findings demonstrate a preventive role for the nuclear actions of gene regulation by PSF and G3BP2 in aging and dementia by sustaining neuronal cell viability.

## 2 RESULTS

### 2.1 PSF expression level is declined in aging

**FIGURE 1:**
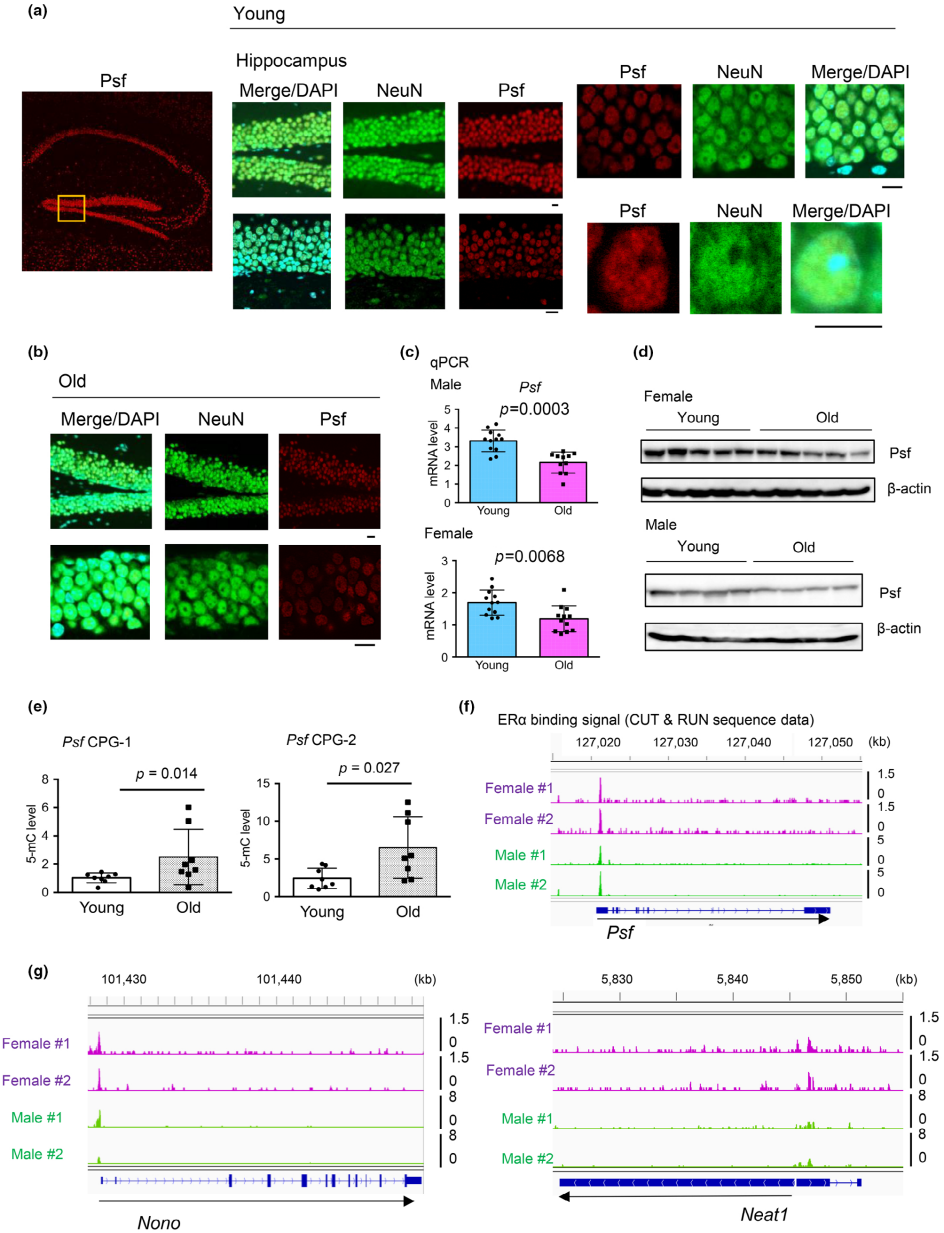
PSF expression level is declined by aging in mouse brain. (a) Representative confocal images of the mouse hippocampus. Immunostaining of Psf and NeuN in young (3 months) mice brains was performed (*N* = 3 for males and females). Bar = 10 μm. (b) Representative confocal images of the hippocampus of old mice. Immunostaining of Psf and NeuN in old (24 months) mouse brains was performed (*N* = 3 for males and females). The Psf signal was reduced compared with that in young mice. Bar = 10 μm. (c) Age‐dependent expression changes of *Psf* mRNA in mouse brain. Quantitative RT‐PCR analysis was performed to measure the mRNA expression of *Psf* in mouse brain samples (hippocampus, *N* = 10–12). The Mann–Whitney *U* test was performed to determine the *p*‐value. Data represents mean ± SD. (d) Age‐dependent changes in Psf protein expression in the mouse brain. Western blotting of Psf in young and old mice is shown (*N* = 5 females and *N* = 4 males). β‐actin was used as loading control. (e) Increased methylation levels around the promoter of *Psf* in old mice compared with young mice Med‐IP was performed using a specific antibody for 5‐mC. DNA samples from the brains of old and young female mice (*N* = 8) were used for the analysis. Two primer sets targeting the CpG regions (CPG‐1 and CPG‐2) around the mouse *Psf* were designed. The Mann–Whitney *U* test was performed to determine the *p*‐value. Data represents mean ± SD. (f) Visualization of ERα binding to *Psf* promoter regions. Publicly available data from CUT and RUN analyses using mouse brain samples are shown (GSE144718). (g) Visualization of ERα binding to promoter regions of other paraspeckle factors (*Nono* and *Neat1*). Publicly available data from CUT and RUN analyses using mouse brain samples are shown (GSE144718).

To explore the potential role of PSF in aging, we first analyzed the expression levels of the Psf protein in young (3 months) and old (24 months) mouse brains. Immunofluorescence (IF) analysis showed that Psf was highly expressed in the nuclei of neurons stained with NeuN in the hippocampi of young mice (Figure 1a). In contrast, we observed that the PSF signal was weaker in old mice than in young mice (Figure 1b). We also investigated PSF mRNA expression levels. Quantitative reverse transcription polymerase chain reaction (qRT‐PCR) showed that *Psf* was significantly reduced at mRNA level in old mice than in young mice (Figure 1c, and Figure S1a). These findings were consistent with the decreased PSF protein levels in the brains of old mice as observed by western blot analysis (Figure 1d). We further investigated the mechanism by which PSF expression is regulated in neurons. Previous reports suggested that altered DNA methylation is involved in the regulation of aging‐associated genes (López‐Otín et al., 2023). Next, we performed methylated DNA immunoprecipitation (Med‐IP) analysis using single‐stranded DNA (ssDNA) fragments containing 5‐methylcytosine (5‐mC) in the brain tissues of young and old mice (Figure 1e and Figure S1c). We observed increased levels of 5‐mC in the promoter regions of *Psf*. Moreover, reduced sex hormone signaling is associated with cognitive dysfunction. Since PSF is involved in the sex steroid hormone signaling pathway (Mitobe et al., 2020; Takayama et al., 2013, 2017), we examined the possibility that PSF expression levels are maintained by sex hormones in the brain. In publicly available data set of global ERα binding sites in mouse brain tissues (Gegenhuber et al., 2022), we showed the recruitment of ERα to the promoter of *Psf*, suggesting the transcriptional regulation of PSF expression by ER in mice (Figure 1f). These findings suggest that age‐associated epigenetic changes and sex hormone levels are important for PSF expression in neurons. We also found that ERα is recruited to the promoter of *Nono* or 5′ upstream regions of *Neat1*, which are other components of paraspeckle and interact with Psf (Figure 1g), suggesting that paraspeckle formation might be mediated by estrogen in the brain of mice.

### 2.2 G3BP2 is highly expressed in hippocampus of mouse and repressed during aging

**FIGURE 2:**
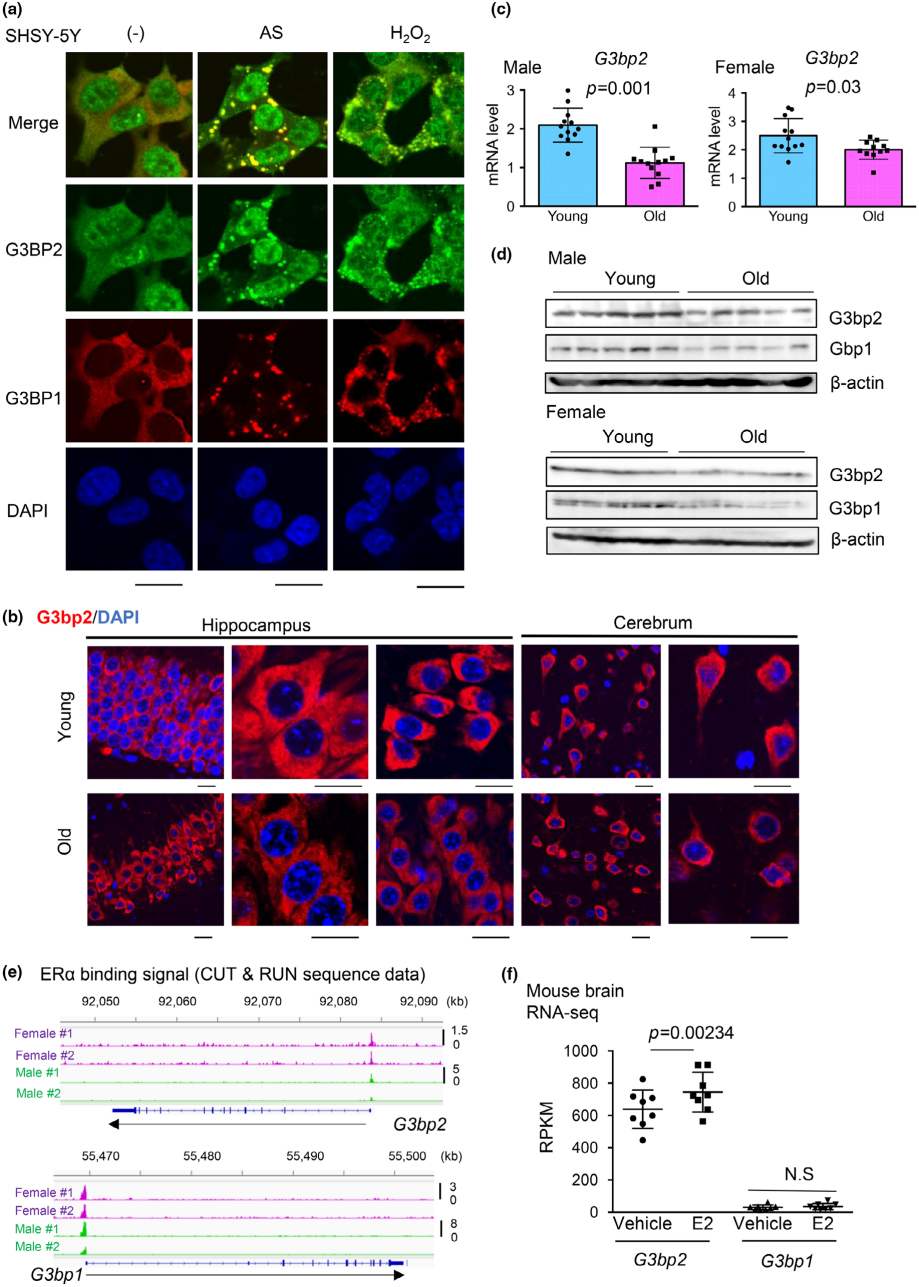
Stress‐granule associated G3BP1/2 expression levels are declined by aging in mouse brain. (a) Immunostaining of G3BP1/2 in SH‐SY5Y cells. Cells were treated with 100 μM sodium arsenite (AS) and 1 mM hydrogen peroxide (H_2_O_2_) for 1 h. Bar = 10 μM. (b) Representative confocal images of the mouse hippocampus and cerebrum. Immunostaining of G3bp2 was performed in young (3 months) and old (24 months) mouse brains (*N* = 3 for males and females). Bar = 10 μm. (c) Age‐dependent expression changes of *G3bp1/2* mRNA in the mouse brain. Quantitative RT‐PCR was performed to measure the mRNA expression of *G3bp1/2* in mouse brain samples (hippocampus, *N* = 12). The Mann–Whitney *U* test was performed to determine the *p*‐value. Data represents mean ± SD. (d) Age‐dependent changes in G3bp1/2 protein expression in the mouse brain. Western blotting of G3BP1/2 in young and old mice (*N* = 5 for males and *N* = 5 or 6 for females). β‐actin was used as loading control. (e) Visualization of ERα binding to *G3bp1*/*2* promoter regions. Publicly available data from CUT and RUN analyses using mouse brain samples are shown (GSE144718). (f) Regulation of *G3bp2* mRNA expression by estrogen in the mouse brain. RNA‐seq data for gene expression in the mouse brain (*N* = 6) were downloaded from (GSE144717). The *p*‐value was calculated using DEseq2. N.S: not significant; RPKM: reads per million mapped reads.

Next, we investigated the expression of another sex hormone‐related RBP, G3BP2, in young and old mouse brains, as changes in G3BP2 expression during aging have not yet been studied. As G3BP1 and G3BP2 are known to be enriched in the SG, we first examined the cellular localization of G3BP2 in human neuronal cells using well‐known oxidative stress inducers, sodium arsenite (AS) and hydrogen peroxide (H_2_O_2_) for stress induction. After stress induction, we observed clear cytoplasmic condensates of G3BP2 in human neuronal cells SH‐SY5Y, colocalizing with another SG marker, G3BP1, suggesting G3BP2 enrichment in SGs in the presence of cell stress (Figure 2a). Notably, G3BP2 expression in the nucleus and high expression levels of G3BP2 compared to those of G3BP1 were observed. We analyzed G3bp2 expression in the mouse brain using immunohistochemistry (IHC). In the brain of young mice, G3bp2 expression was predominantly detected in the cytoplasm of neurons in the hippocampus and cerebrum. However, no SG foci were observed in the brain. Next, we analyzed whether G3BP2 expression changed with age. We observed a reduced signal in the neurons of the old mouse brains (Figure 2b). qRT‐PCR analysis showed a significant decrease in the mRNA level of *G3bp2* in the hippocampus of old mice compared to young mice (Figure 2c, and Figure S1b). Western blot analysis also demonstrated a reduction in G3bp1/2 protein levels in the brains of old mice compared to young mice (Figure 2d). Overall, these results indicate that G3BP2 expression decreases during aging. We then investigated the molecular mechanisms underlying decreased G3BP2 expression levels in neurons. We previously reported androgen‐mediated regulation of G3BP2 expression by recruiting AR to the promoter region (Ashikari et al., 2017). In mouse brain tissues, androgen exhibited its activity by being converted to estrogen by tissue specific aromatase (Wu et al., 2009). Therefore, we investigated whether G3bp2 is regulated by estrogen in the mouse brain. Interestingly, CUT&RUN data showed ERα binding to the promoter regions of *G3bp1/2* in both male and female mice (Figure 2e). RNA sequencing (RNA‐seq) data indicated significant upregulation of G3BP2 expression by ER and a robustly higher expression of *G3bp2* than *G3bp1* in the mouse brain (Figure 2f). Collectively, these results demonstrate that G3BP2 is the predominant subtype of G3BPs in the brain and that its expression is induced by sex hormones.

Furthermore, we analyzed the differences between male and female mice in the reduction of Psf and G3bp2 expression levels, as well as other proteins, Nono and G3bp1, in the mouse brain using western blot analysis. In both the cerebrum and hippocampus, we observed a reduction in G3bp1/2, Psf, and Nono expression levels in old mice compared to young mice. Interestingly, this change in expression change of G3bp2, Psf, and Nono was more evident in the hippocampus, especially in female mice, whereas we observed a more marked G3bp1 reduction in the cerebrum than in the hippocampus (Figure S1d). Thus, this analysis suggests sex‐dependent and brain region‐specific expression changes in Psf and G3bp2 during aging.

### 2.3 PSF interacts with G3BP2 predominantly in nucleus to sustain neuronal cell viability

**FIGURE 3:**
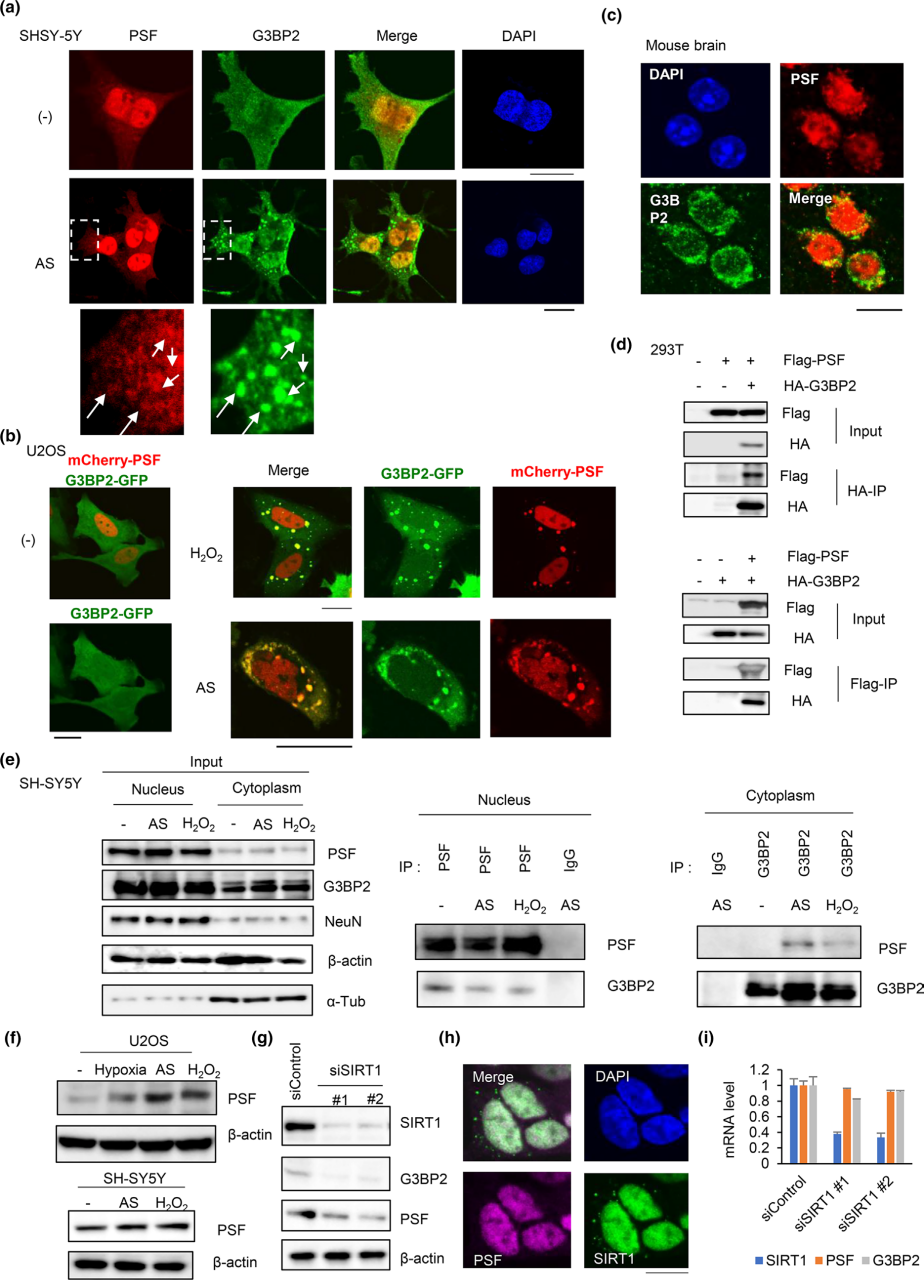
PSF interacts with G3BP2 predominantly in the nucleus of neuronal cells. (a) Immunostaining of PSF and G3BP2 in SH‐SY5Y cells. Cells were treated with 100 μM AS for 1 h. Bar = 10 μM. Arrows indicate cytoplasmic granules containing G3BP2 and PSF. (b) Colocalization of transfected PSF and G3BP2 proteins in both the nucleus and stress granules. U2OS cells were transfected with GFP‐G3BP2 and mCherry‐PSF. After 24 h incubation, cells were treated with 100 μM AS or 1 mM H_2_O_2_ for 1 h, and then observed by confocal microscope. Bar = 10 μm. (c) Immunostaining for G3BP2 and PSF was performed on the mouse brain (3 months male, cerebrum). Bar = 10 μm. (d) 293 T cells were transfected with Flag‐PSF and HA‐G3BP2 as indicated. After 24 h of incubation, the cells were harvested for immunoprecipitation using anti‐Flag or anti‐HA antibodies. Western blotting was performed to evaluate protein expression. (e) Interaction between G3BP2 and PSF in the nuclei of neurons SH‐SY5Y cells were treated with 100 μM AS or 1 mM H_2_O_2_ for 1 h. Cell lysates from the nuclear and cytoplasmic fractions were immunoprecipitated with anti‐G3BP2, anti‐PSF, or nonspecific IgG. Western blotting was performed to evaluate protein expression. (f) Western blot analysis of PSF in U2OS and SH‐SY5Y cells. Both cells were treated with 100 μM AS or 1 mM H_2_O_2_ for 1 h before harvesting. β‐actin was used as loading control. (g) PSF and G3BP2 expressions were positively regulated by SIRT1 in neuronal cells. SH‐SY5Y cells were treated with siSIRT1 #1 and #2 or siControl (10 nM) for 48 h. Western blotting was performed to detect SIRT1 and PSF. β‐actin was used as loading control. (h) Immunostaining of SIRT1 and PSF in SH‐SY5Y cells. Bar = 10 μM. (i) SH‐SY5Y cells were treated with siSIRT1 #1 and #2 or siControl (10 nM) for 48 h. qRT‐PCR was performed to evaluate the expression of SIRT1, G3BP2, and PSF. Data represents mean ± SD.

Recent studies have linked PSF to the regulation of neuronal differentiation in mice by modifying transcribed pre‐mRNAs. In this study, we analyzed whether PSF‐mediated gene regulation was associated with G3BP2 function in the nucleus. In endogenous human neuronal cells expressing these proteins (SH‐SY5Y and NB1), IF analysis showed predominant enrichment of G3BP2 and PSF in the nucleus (Figure 3a and Figure S2a). Interestingly, we also detected cytoplasmic interactions between PSF and G3BP2‐enriched granules after stress induction, which is in line with a previous report that PSF could be enriched in SGs in HeLa cells (Younas et al., 2020). We then analyzed the translocation of PSF into SGs by overexpressing PSF and its cotransfection with G3BP2 in U2OS cells (Figure 3b). We detected a clear translocation of PSF to G3BP2‐enriched granules following stress treatment. This translocation was dependent on the N‐terminal region of PSF, suggesting that the low‐complexity domain of PSF is important for its translocation and enrichment in SGs (Figure S3a,b). In the mouse brain, we observed PSF immunopositivity primarily in the nucleus and G3BP2 immunopositivity in the cytoplasm, with an overlap in the nuclear and perinuclear regions (Figure 3c). Co‐immunoprecipitation of transfected G3BP2 and PSF showed a mutual interaction between these proteins (Figure 3d). We then analyzed the interaction of PSF with G3BP2 by separating the cells into nuclear and cytosolic fractions. High levels of PSF and G3BP2 were detected in the nucleus. The co‐immunoprecipitation assay demonstrated an interaction in the nucleus, both with and without stress induction (Figure 3e and Figure S2b). In contrast, a slight increase in the interaction in the cytoplasm was detected, suggesting the enrichment of PSF in the SGs following stress treatment. Consistent with a previous report (Younas et al., 2020), western blot analysis of whole‐cell lysates indicated stress‐induced induction of PSF in SH‐SY5Y and U2OS cells (Figure 3f). Furthermore, an in vitro binding assay with purified G3BP2 and PSF showed that this interaction was direct (Figure S2c). The use of deletion proteins of G3BP2 and PSF revealed the importance of the C‐terminal region (326–483 a.a) of G3BP2 and the RNA‐binding domain of PSF for the interaction (Figure S2d,e and Figure S3c). Since the C‐terminal region includes the RNA‐binding domain of G3BP2, the RNA‐binding domains of both proteins could be important for this interaction.

Moreover, sirtuin 1 (SIRT1)‐mediated modification are important for longevity and aging (Oberdoerffer et al., 2008). It is well known that SIRT1 expression is repressed during aging. Therefore, we examined whether SIRT1 is involved in the expression of PSF and G3BP2 using western blotting and IF analyses in human neuronal SH‐SY5Y cells. Interestingly, SIRT1 knockdown resulted in reduced expression of PSF and G3BP2 (Figure 3g) and the co‐localization of PSF and SIRT1 in the nucleus (Figure 3h). However, the mRNA levels of PSF and G3BP2 were not significantly affected by SIRT1 knockdown, suggesting that deacetylation of the PSF protein is important for the expression of PSF and G3BP2 (Figure 3i).

**FIGURE 4:**
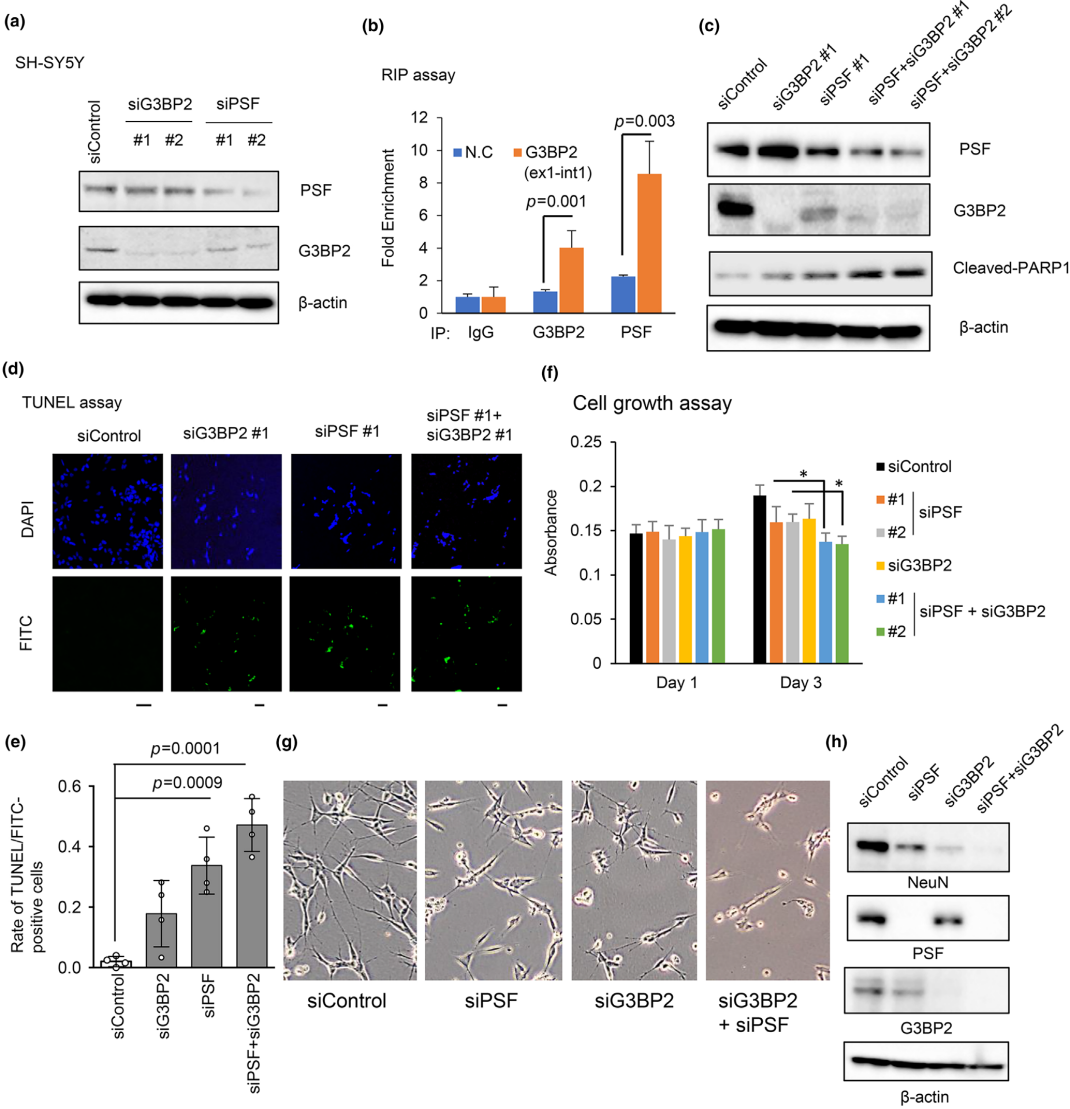
Induction of apoptosis by silencing PSF and G3BP2 in neuronal cells. (a) Silencing G3BP2 and PSF expression in neuronal cells. SH‐SY5Y cells were treated with siG3BP2 #1 and #2 (5 nM), siPSF #1 and #2 (10 nM), or siControl, as indicated. The protein expression levels of G3BP2 and PSF were evaluated using western blot analysis. β‐actin was used as loading control. (b) Interaction of G3BP2 and PSF proteins with G3BP2 pre‐mRNA RNA immunoprecipitation (RIP) assay for G3BP2 and PSF was performed on SH‐SY5Y cells (*N* = 3). Normal IgG served as a negative control. *N*.*C*.: Negative control locus (*GAPDH exon1*‐ *intron1*). A two‐sided *t* test was performed to determine *p*‐values (vs. *N*.*C*.). Data represents mean ± SD. (c) Silencing G3BP2 and PSF expression induces apoptosis in neuronal cells. SH‐SY5Y cells were treated with siG3BP2 #1, #2 (5 nM), siPSF #1 (10 nM), or siControl for 48 h. The protein expression levels of cleaved PARP1, G3BP2, and PSF were evaluated using western blot analysis. β‐actin was used as loading control. (d) TUNEL assay of neuronal cells after silencing G3BP2 and PSF. Representative images of FITC‐positive SH‐SY5Y cells treated with siG3BP2 #1 and #2 (5 nM), siPSF #1 and #2 (10 nM), or siControl for 48 h. (e) Quantification of TUNEL‐positive cells (*N* = 4 biological replicates). One‐way ANOVA and Tukey's post‐hoc tests were performed to obtain *p*‐values (vs. siControl). Data represents mean ± SD. (f) Cell proliferation assay of neuronal cells. SH‐Y5Y cells were transfected with siControl, siPSF #1, siPSF #2 (10 nM), or siG3BP2 #1 (5 nM). After 72 h incubation, the MTS assay was used to quantify cell growth rate (*N* = 4, biologically independent). *p < 0.05. Two‐tailed *t* tests were performed. Data are presented as mean ± SD. (g) Microscopic images of neuronal cells incubated in differentiation medium. SH‐SY5Y cells were treated with siControl, siPSF #1 (10 nM), or siG3BP2 #1 (5 nM) as indicated. The cells were incubated in differentiation medium for 5 days. (h) Immunoblotting of PSF, G3BP2, and NeuN in SH‐SY5Y cells transfected with siControl, siPSF #1 (10 nM), or siG3BP2 #1 (5 nM). β‐actin was used as loading control. The cells were incubated in differentiation medium for 5 days.

SG‐mediated attenuation of translation prevents apoptosis and sustains cell viability. Therefore, we investigated the effects of PSF and G3BP2 knockdown on human neuronal cells (Figure 4a, and Figure S4a,b). Interestingly, PSF knockdown reduced G3BP2 mRNA and protein levels. To investigate the PSF‐mediated regulatory mechanism of G3BP2, RNA immunoprecipitation (RIP) assay was performed. Amplification of regions spanning exon1‐intron1 junctions using IP PSF‐ or G3BP2‐RNAs and efficiencies of enrichment relative to the background amount associated with the control IgG were analyzed. The qRT‐PCR results showed an interaction between PSF and the pre‐mRNA of G3BP2 (Figure 4b), suggesting the regulation of G3BP2 by PSF at the RNA level. In this study, we explored the effects of PSF and G3BP2 knockdown on neuronal cell viability. Western blot analysis showed a clear induction of the apoptosis marker, cleaved PARP1, by knockdown of both PSF and G3BP2 (Figure 4c and Figure S4c). Furthermore, we evaluated the effects of PSF and G3BP2 knockdown on apoptosis induction using a terminal deoxynucleotidyl transferase dUTP nick‐end labeling (TUNEL) assay. We observed that the knockdown of each gene, and more dramatically, both genes, induced apoptosis in SH‐SY5Y cells (Figure 4d,e). In addition to their anti‐apoptotic effects, knockdown of PSF and G3BP2 inhibits cell proliferation (Figure 4f). We further induced the differentiation of neuronal SH‐SY5Y cells from an initial epithelial‐like cell phenotype to a more expansive and branching neuronal phenotype by culturing the cells in medium containing retinoic acid (RA). We found that knockdown of PSF and G3BP2 reduced the number of cells with a branching phenotype, characterized by obvious neurite and axon formation (Figure 4g). We examined the expression of NeuN, a representative neuronal marker. NeuN expression in differentiation medium was suppressed by knockdown of PSF and G3BP2 (Figure 4h), suggesting an important role of PSF and G3BP2 in neuronal differentiation. Overall, these results demonstrated that the cooperative action of PSF and G3BP2, particularly in the nucleus, prevented apoptosis, and maintained neuronal differentiation and cell viability.

Moreover, we investigated whether another paraspeckle factor *NEAT1* has a cooperative effect on PSF‐ and G3BP2‐mediated functions in neuronal cells. We repressed *NEAT1* expression using siRNAs targeting *NEAT1* (Figure S5a). After *NEAT1* knockdown, increased cell growth was observed (Figure S5b). Furthermore, the oxidative stress induced by H_2_O_2_ treatment triggered DNA damage response and cell apoptosis in SH‐SY5Y cells. We found that *NEAT1* knockdown alleviated these stress responses, suggesting that *NEAT1* plays a role in promoting the oxidative stress‐mediated damage response in neuronal cells, in contrast to the preventive role of PSF and G3BP2 (Figure S5c). Consistently, RNA‐seq data in mouse brain suggest that ER, which plays an antioxidant role in the brain (Behl, 2002), can repress the expression level of *NEAT1* in the mouse brain (Figure S5d). Thus, *NEAT1* plays a different role than PSF and G3BP2 in neuronal cell activity.

### 2.4 PSF and G3BP2 cooperatively regulates their target genes associated with neuron activity and AD progression

**FIGURE 5:**
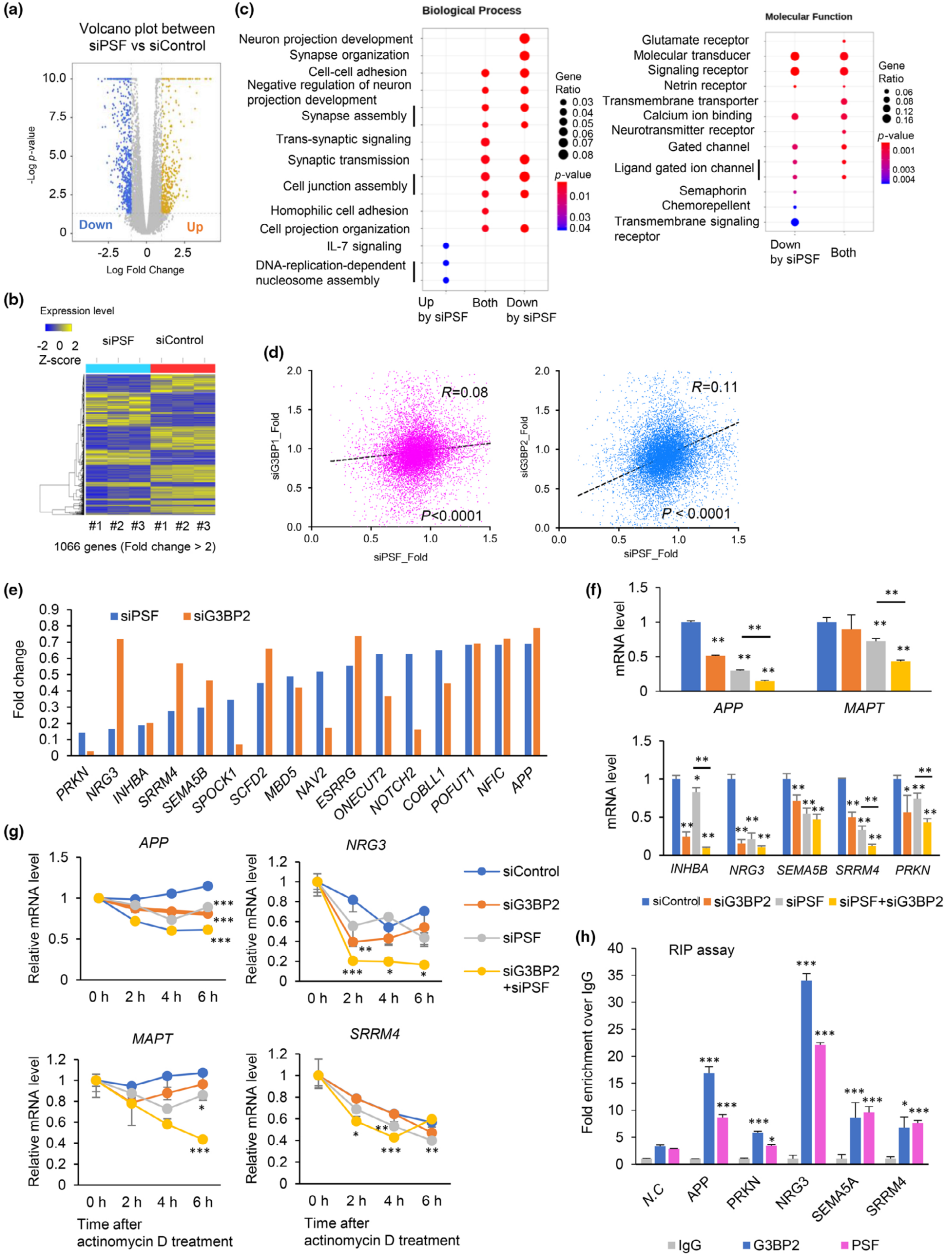
PSF and G3BP2 cooperatively sustain the expression of genes associated with neuronal activity. (a) PSF silencing inhibited neuronal activity‐associated genes. SH‐SY5Y cells were treated with siControl or siPSF #2 (10 nM) for 72 h. Total RNA was extracted for RNA‐seq analysis (*N* = 3, biological triplicates). A volcano plot of the RNA‐seq data used to visualize significant genes is shown. (b) Heatmap showing down‐ or upregulated genes (fold change >2: Total of 1066 genes) following PSF knockdown in SH‐SY5Y cells. (c) GO term analysis showing changes in hallmark pathways. GO term analysis showing enrichment of neuron‐associated biological processes or molecular functions among genes downregulated by PSF knockdown based on RNAseq in SH‐SY5Y cells. (d) Positive correlation between G3BP2‐ and PSF‐regulated gene expression We used previously reported RNA‐seq data (Sato et al., 2023) showing G3BP1‐ and G3BP2‐regulated genes in SH‐SY5Y cells. Fold changes relative to the siControl were used for evaluation. A regression analysis was performed to analyze the correlations. (e) The expression levels of representative genes in SH‐SY5Y cells treated with siPSF #2 and siG3BP2 #1 relative to siControl are shown. The top 15 genes downregulated by PSF knockdown were selected using RNA‐seq data. APP was used as a positive control. (f) SH‐SY5Y cells were treated with siControl, siPSF #2, or siG3BP2 #1, as indicated. Total RNA was extracted, and qRT‐PCR was performed to analyze the expression level of G3BP2/PSF‐target genes at the mRNA level (*N* = 3). A two‐sided *t* test was performed to determine the p‐value (vs. siControl). Data represents mean ± SD. (g) Stability of G3BP2/PSF‐target transcripts in SH‐SY5Y cells treated with siPSF, siG3BP2, or siControl. Cells were incubated with 2 μg/mL actinomycin D for the indicated times, and target RNA quantities at different time points were evaluated by qRT‐PCR (*N* = 3). A two‐sided *t* test was performed to determine the *p*‐value (vs. siControl). Data represents mean ± SD. (h) Interaction of G3BP2 and PSF with the pre‐mRNA of G3BP2/PSF‐target genes. RNA immunoprecipitation (RIP) assay for G3BP2 and PSF was performed on SH‐SY5Y cells. Pre‐mRNA levels were evaluated using qRT‐PCR (*N* = 3). Normal IgG was used as a negative control. N.C.: Negative control locus (*GAPDH exon1*‐ *intron1*). A two‐sided *t* test was performed to determine *p*‐values (vs. N.C.). Data represents mean ± SD. *p < 0.05, **p < 0.01, ***p < 0.001.

In a previous study, we performed a global analysis of the G3BP1/2‐mediated regulation of RNAs (Sato et al., 2023). We then found that AD‐associated gene transcripts accumulated among RNAs regulated by G3BP1/2, suggesting a role for G3BP2‐mediated control of RNAs in AD development (Sato et al., 2023). To investigate whether there is a cooperative relationship between gene regulation and neuronal cell activity mediated by PSF and G3BP2, we performed RNA‐seq analysis of PSF target genes in human neuronal cells SH‐SY5Y (Figure 5a). Notably, PSF knockdown resulted in the downregulation of genes involved in critical neuronal functions such as receptors and synaptic activity (Figure 5b,c). We then compared the siPSF‐mediated repression of gene expression with that of siG3BP1/2. A significant positive correlation was detected between the PSF‐ and G3BP2‐regulated genes, suggesting cooperative gene regulation by PSF and G3BP2 (Figure 5d).

We further analyzed the regulatory mechanisms of PSF and G3BP2. We focused on representative target genes that were positively regulated by PSF and G3BP2 (Figure 5e). Previous reports have documented the post‐transcriptional regulation of *APP* and *MAPT*/*TAU* (Ray et al., 2011; Takayama et al., 2019), indicating the alternative splicing of these genes in AD development. In addition, RNA‐seq analysis has identified the cooperative effects of PSF and G3BP2 knockdown on several genes, including *INHBA* (Lau et al., 2015; Oberländer et al., 2022), *NRG3* (Wang et al., 2014), *SEMA5B* (Matsuoka et al., 2011; Seto et al., 2022), *SRRM4* (Chung et al., 2018) and *PRKN* (Watzlawik et al., 2023). Interestingly, according to previous studies, the loss of these genes is associated with neuronal activity and AD progression. qRT‐PCR analysis confirmed that these genes were cooperatively regulated by PSF and G3BP2 in several human neuronal cell lines (Figure 5f, and Figure S4d,e). In addition, we analyzed whether other paraspeckle factors are involved in gene regulation. We used siRNAs targeting NONO to reduce its expression levels (Figure S4f). However, no significant changes in gene expression were observed, suggesting that cooperative gene regulation by PSF and G3BP2 is independent of other paraspeckle factors. We also analyzed the positive effects of co‐overexpression of G3BP2 and PSF on the expression of these target genes in U2OS cells. We observed increased APP expression at both the protein and mRNA levels following co‐transfection with PSF and G3BP2 (Figure S6a,b).

We further assessed the effects of PSF and G3BP2 on the stability of the target mRNA by blocking new RNA synthesis following actinomycin D treatment. The results of qRT‐PCR analysis showed that the mRNA stability of these target genes was significantly decreased in knockdown cells of both PSF and G3BP2 compared to control cells (Figure 5g and Figure S6c), indicating the role of these RBPs in the post‐transcriptional stabilization of mRNA expression. Moreover, the interaction between the pre‐mRNA and G3BP2 and PSF proteins was examined using the RIP assay (Figure 5h and Figure S6d). *APP* exon1‐intron1 amplification was used as a positive control for PSF‐associated RNA (Takayama et al., 2019). Different regions of the tested transcripts were efficiently amplified, suggesting that pre‐mRNAs were associated with PSF and G3BP2 for post‐transcriptional regulation. Collectively, our data demonstrate that PSF and G3BP2 cooperatively stabilize the target mRNA by directly binding to its pre‐mRNA.

### 2.5 Reduced expression of G3BP2 and PSF in nuclei of neurons in AD brain

**FIGURE 6:**
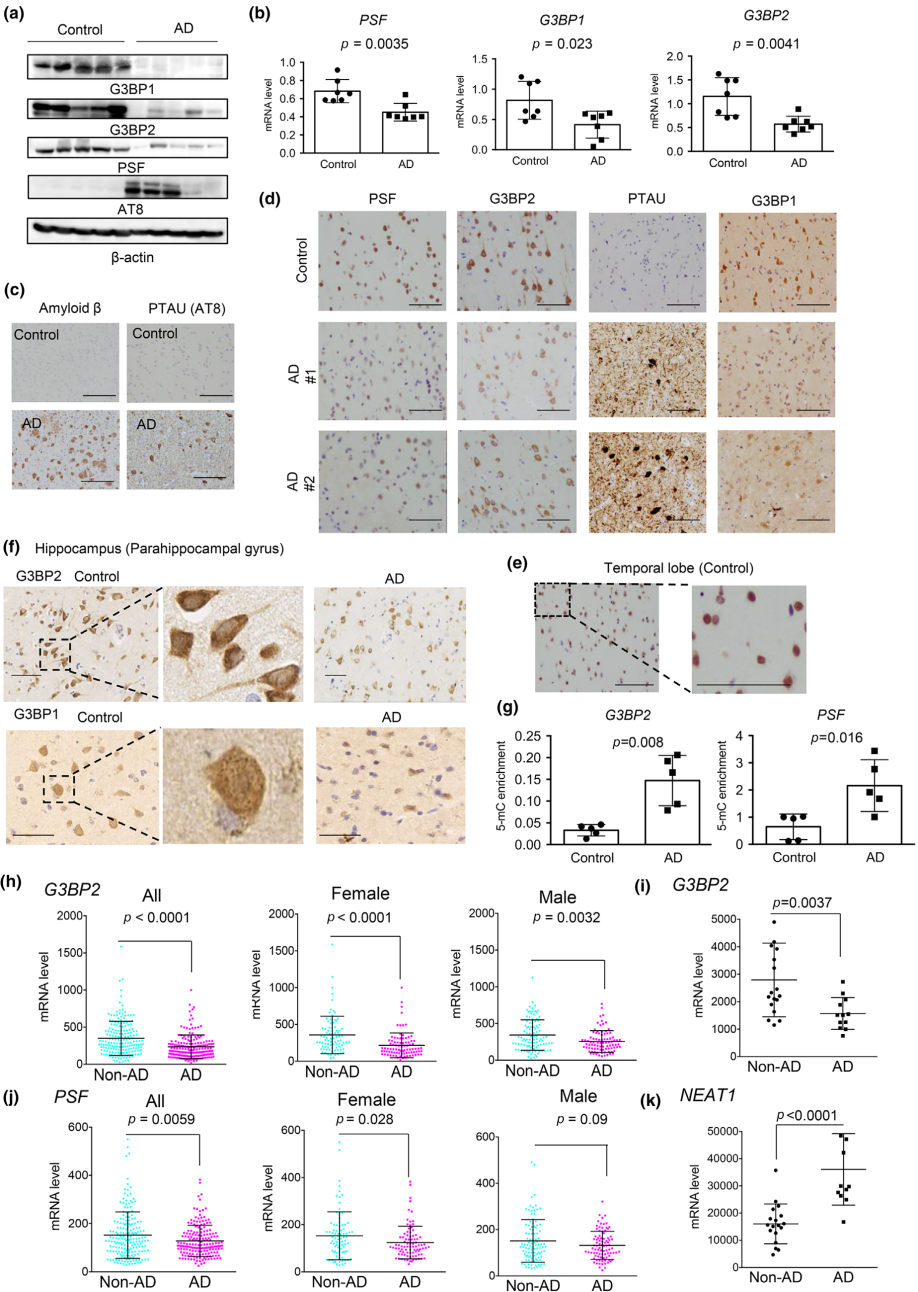
Reduced expression of PSF and G3BP2 in human AD brain. (a) Representative immunoblot images showing expression of G3BP1/2, PSF, and p‐tau (AT8). Western blot analysis was performed using temporal brain tissue from patients with AD and non‐AD controls (*N* = 5). β‐actin was used as loading control. (b) The mRNA expression levels of G3BP1/2 and PSF at mRNA level was analyzed by qRT‐PCR in AD and non‐AD controls (*N* = 7). The Mann–Whitney *U* test was performed to determine the *p*‐value. Data represents mean ± SD. (c) Immunohistochemistry was performed on temporal brain tissues from patients with AD and non‐AD controls (*N* = 5). Representative images of Amyloid β and PTAU (AT8) are shown. (d) The representative images of PSF, G3BP1/2, and PTAU (AT8) are shown (*N* = 5). (e) Immunohistochemical analysis of PSF in temporal human brain tissue (non‐AD controls). Nuclear enrichment of PSF was observed. Bar = 50 μm. (f) Nuclear localization of G3BP1/2 in the human brain tissue. Hippocampal brain tissues from AD patients (*N* = 6) and non‐AD controls (*N* = 4) were stained for G3BP1/2. Magnified images show nuclear expression of G3BP1/2 and cytoplasmic granules of G3BP1/2 in non‐AD controls. Bar = 50 μm. (g) Increased methylation levels around the G3BP2 promoter and PSF in brain tissues from AD and non‐AD controls. Med‐IP was performed using a specific antibody for 5‐mC. DNA samples (*N* = 5 per group) were used for the analysis. The Mann–Whitney *U* test was performed to determine the *p*‐value. Data represent mean ± SD. (h) Expression level of *G3BP2* in human brain from AD patients (*N* = 176, female: 88, male: 88) or control group (non‐AD) (*N* = 187, female: 85, male: 102). A large transcriptome dataset (GSE15222) was used for this study. An unpaired *t* test was used to determine *p*‐values. Data represent mean ± SD. (i) Expression level of *G3BP2* in the brain of patients with AD (*N* = 12) or controls (non‐AD) (*N* = 18). RNA‐Seq data (GSE159699) was used. The Mann–Whitney *U* test was performed to determine *p*‐values. Data represent mean ± SD. (j) Expression levels of *PSF* in the brain of AD patients (*N* = 176, female: 88, male: 88) and control (non‐AD) (*N* = 187, female: 85, male: 102). A large transcriptome dataset (GSE15222) was used for this study. An unpaired t‐test was used to determine *p*‐values. Data represent mean ± SD. (k) Expression level of the paraspeckle factor *NEAT1* in the brains of patients with AD (*N* = 12) or controls (non‐AD) (*N* = 18). RNA‐Seq data (GSE159699) was used. The Mann–Whitney *U* test was performed to determine the *p*‐values. Data represent mean ± SD.

Next, we evaluated PSF and G3BP1/2 expression in patients with sporadic AD and in controls (*N* = 5). Representative western blot results showed that the levels of these RBPs were significantly decreased in patients with AD, although the deposited amounts of phosphorylated tau proteins increased (Figure 6a). In addition, we performed qRT‐PCR to investigate the expression levels and observed a similar downregulation in AD samples at the mRNA level (Figure 6b). To examine the cellular localization of PSF and G3BP1/2 in sporadic AD, we performed IHC using brain tissues from patients with AD and controls (Figure 6c–f). In temporal lobes of AD brains, we detected Aβ and phosphorylated TAU (PTAU) accumulation in brains (Figure 6c). Nuclear staining of the PSF protein was alleviated in AD samples compared to controls, in line with previous reports and western blot analyses (Figure 6d). We detected PSF immunoreactivity in the nuclei of both the AD and control brains (Figure 6e). However, dyslocalization of PSF (Ke et al., 2012) was not observed in this study. In the human brain, including the temporal lobe and hippocampus, we detected nuclear and cytoplasmic localization of G3BP1/2 expression (Figure 6d,f). These results showed that PSF colocalized with G3BP2 in the nuclei of human neuronal cells. Interestingly, G3BP1/2‐foci foci were detected in the cytoplasm, suggesting SG formation in the normal human brain (Figure 6f). We also observed diminished G3BP1/2 reactivity in the AD brains (Figure 6d,f). Notably, reduced expression of G3BP1/2 or aggregation‐like structures was observed in AD brains, along with PTAU aggregation (Figure S7a–d). Furthermore, Med‐IP analysis demonstrated that DNA methylation of promoters at both PSF and G3BP2 may be involved in the downregulation of these genes in AD samples (Figure 6g).

We also analyzed the expression levels of PSF, G3BP2, and other paraspeckle factors using publicly available transcriptome data (GSE15222 and GSE159699). Consistent with our results, the transcriptome data showed that the expression levels of PSF and G3BP2 were lower in human AD samples than in non‐AD samples (Figure 6h–j). However, the expression levels of NONO in AD patients did not change compared to those in non‐AD patients (Figure S7e). Interestingly, this decrease in expression was more pronounced in women than in men, suggesting an involvement of PSF and G3BP2 in AD pathology, particularly in women. On the contrary, another paraspeckle factor *NEAT1* was significantly higher in AD samples than in non‐AD samples in RNA‐seq data (Figure 6k). Taken together, these clinical data suggest that the nuclear activity of PSF and G3BP2, independent of paraspeckle formation, may be involved in the AD development and progression.

## 3 DISCUSSION

RBPs regulate gene expression at epigenetic, splicing, and translational levels. These functions are essential for neuronal cell growth and differentiation (Wolozin & Ivanov, 2019). Here, we provided evidence that PSF and G3BP2 cooperatively mediate neuronal activity and AD‐related gene expression at the post‐transcriptional level by binding to pre‐mRNAs. Typically, RBPs include a low‐complexity domain in their sequence that regulates self‐aggregation (Cao et al., 2020). This regulated aggregation is critical for RBP function, because numerous RBPs facilitate the formation of various ribonucleoprotein (RNP) granules including paraspeckles and SGs. SGs are responsible for the transient recruitment of mRNA transcripts and aggregation in the cytoplasm (Guillén‐Boixet et al., 2020). Although G3BP2 is an important component of SGs, identification of the interaction between PSF and G3BP2 primarily in the nucleus suggests that G3BP2 plays a functionally important role in neurons associated with human neuronal diseases. Importantly, we provide the first evidence that G3BP2 expression decreases during AD development, along with PSF.

Interestingly, recent evidence suggests that RBPs included in SG components, such as TIA‐1, co‐localize with hyperphosphorylated tau (Vanderweyde et al., 2016). TIA1 accumulation is observed in tau pathology of diseases such as AD and FTD (Vanderweyde et al., 2012). The association of TIA‐1 with tau also promotes the tau‐mediated degeneration of primary hippocampal neurons. In addition, cellular stress treatment enhances the enrichment of phosphorylated tau in the SGs (Maziuk et al., 2018). Reduced TIA‐1 expression rescues cognitive decline and increases survival in a mouse model of tauopathy (Vanderweyde et al., 2016). Thus, the high concentration of RNA and RBPs included in SGs raises the possibility that SG formation may lead to tau aggregation to assemble complexes of paired helical filaments (PHFs). In contrast, a recent study showed that G3BP2 interacts with the tau protein and prevents tau aggregation in the brain (Wang et al., 2023). Loss of G3BP2 increased tau pathology in human AD organoids. Moreover, we showed that G3BP2‐assciated RNAs in SGs include signals related to AD development (Sato et al., 2023). In the present study, we demonstrated that G3BP2 is the main subtype of the G3BP family in the brain. G3BP2 regulates target gene expression by associating with PSF in the nucleus. In our clinical analysis, G3BP2 expression levels were reduced in the brains of patients with AD compared with controls. Taken together, these findings suggest that gene regulation at the RNA level as well as G3BP2‐mediated inhibition of tau accumulation at the protein level are important for sustaining cell viability and preventing AD development.

This study also demonstrated that PSF and G3BP2 expression decreased during aging in mice at both the RNA and protein levels. Mechanistically, Med‐IP and qRT‐PCR analyses revealed that changes in DNA methylation patterns during AD progression and aging are important for this repression (López‐Otín et al., 2023). In addition to other molecular mechanisms that repress expression, we observed ERα recruitment to the promoters in mouse brains. In addition to PSF and G3BP2, we found that ERα targets other paraspeckle factors such as NONO and *NEAT1* as well as another subtype of the G3BP family, G3BP1, raising the possibility that both paraspeckles, and SGs are modulated by ER in the brain. In previous studies, we reported that PSF‐dependent gene regulation is important for ERα and AR expression by regulating them at the post‐transcriptional level. We also reported that androgens induce G3BP2 expression. Animal studies have shown that estrogens are converted from androgens by brain‐specific aromatase and play an important role in brain activity, in male mice (Wu et al., 2009). As serum concentrations of sex hormones such as androgens and estrogens decline during aging, reduced ER signaling may contribute to the transcriptional repression of these RBPs. We showed that protein levels of PSF and G3BP2 changed more markedly in the hippocampus than in the cerebrum of female mice during aging, suggesting differential regulation of brain PSF and G3BP2 expression between males and females. Differences in the effects of sex hormone between male and female mice may be involved in the sex‐specific regulation of protein expression. Furthermore, we observed that SIRT1 interacted with PSF and that SIRT1 knockdown reduced PSF expression. SIRT1 delays aging and extends the lifespan of mice (Oberdoerffer et al., 2008). The deacetylase activity of SIRT1 rescues cell survival and mitochondrial function, and prevents premature aging. Decreased expression of SIRT1 been observed in various geriatric diseases, such as ALS and AD (Kim et al., 2007). Thus, SIRT1‐mediated deacetylation may be necessary to maintain PSF expression at the protein level, although it remains to be determined whether direct modulation of PSF protein by SIRT1 or an indirect pathway mediated by SIRT1 is responsible for this regulation.

Consistent with our results, recent evidence has shown that PSF is dysregulated in the postmortem brains of patients with AD (Ke et al., 2012; Younas et al., 2020). Furthermore, PSF mutations have been identified in the motor neuron disease ALS (Hennig et al., 2017). Moreover, when PSF is knocked down, mice exhibit FTD‐like behavior, with the accumulation of phosphorylated tau through the dysfunction of alternative splicing, reduced adult neurogenesis, and neuronal loss in the hippocampus (Ishigaki et al., 2017). Inhibition of PSF‐mediated epigenetic regulation and splicing activity would modulate the downstream signaling pathway including APP and tau expression and isoform changes that contribute to Aβ generation and tau aggregation (Coronel et al., 2018; Lee et al., 2018). Consistent with previous reports, we observed that PSF is predominantly expressed in the human brain nucleus and is reduced in AD brains compared to controls. Although the cytoplasmic expression of PSF in ALS neurons and AD brains has been previously reported (Younas et al., 2020), we did not observe PSF dislocations in the cytoplasm of AD tissues in our IHC analysis. Interestingly, SG‐like structures in the cytoplasm of neurons in human brains and aggregation‐like structures in the brains of patients with AD were observed in our G3BP2 IHC analysis, consistent with the central role of G3BP2 in SG formation (Cao et al., 2020; Guillén‐Boixet et al., 2020). Further IF analyses of the functional structure, accumulation, and cellular localization of PSF and G3BP2 in AD brain tissue would be helpful for understanding the additional roles of RBPs in AD development. Furthermore, using extensive transcriptome data, we confirmed that both PSF and G3BP2 were significantly downregulated in AD compared to normal human brains. This decline was more evident in women than in men. Similarly, sex differences in gene expression in AD tissues have been observed in RNA‐Seq studies (Shulman et al., 2023). We hypothesize that sex differences in the effects of estrogen and androgen are reflected in the reduction in the expression of these proteins. These differences may be related to cognitive decline or AD pathologies.

Our RNA‐seq analyses showed that PSF targets various genes related to AD progression (Chung et al., 2018; Lau et al., 2015; Wang et al., 2014; Seto et al., 2022). Therefore, our results suggest that repression of PSF‐mediated signaling inhibits the signaling pathways involved in neuronal activity. In addition to the signals associated with functional neuronal activity, other important PSF targets at the RNA level include *G3BP2*, *APP*, and *Tau*/*MAPT*. We found that G3BP2 expression was maintained by PSF binding to the G3BP2 pre‐mRNA. This finding indicated that the regulatory network of RBPs promotes their expression at the RNA level. In addition, we identified target genes of PSF and G3BP2 using RNA‐Seq analysis. Interestingly, genes associated with neuronal activity were enriched among the target genes. However, in the present study, we were unable to identify genes associated with metabolic changes and lipid biology, identified as important signals in AD by RNA‐seq analysis recently (Barbash et al., 2017). For example, among the identified target genes in our analysis, SEMA5B maintains axon growth during nervous system development. This protein product can be cleaved to function as a secretory molecule (Matsuoka et al., 2011). SEMA5B is downregulated in AD and associated with Aβ deposition (Seto et al., 2022). Therefore, impaired expression of these target genes would inhibit neuronal function and cognition. We hypothesize that upregulation of the expression levels of these target genes contributes to the maintenance of neuronal activity in the brain through the cooperative action of PSF and G3BP2. Mechanistically, both PSF and G3BP2 have RNA‐binding domains that regulate RNA processing of their target genes. The mRNA stability of these target genes could be suppressed by the knockdown of PSF and G3BP2, suggesting the role of these RBPs in the post‐transcriptional stabilization of mRNA expression. Moreover, we demonstrated that PSF and G3BP2 bind to the pre‐mRNAs of target genes at the RNA level by performing an RNA immunoprecipitation assay. We set primers against the intron‐exon regions of these target genes to detect pre‐mRNAs that show regulation of target genes at the post‐transcriptional level to promote RNA splicing. Overall, our results suggest that nuclear signaling mediated by PSF and G3BP2 is important to prevent AD development through the post‐transcriptional regulation of important genes involved in neuronal activity.

Furthermore, we examined other paraspeckle factors associated with PSF and G3BP2 signaling. *NEAT1* is a long non‐coding RNA that is enriched in the nucleus and is essential for the nuclear paraspeckle formation (Clemson et al., 2009). Paraspeckles play an important role in many gene regulation processes, such as mRNA retention, A‐to‐I editing, and protein sequestration. *NEAT1* serves as a platform for the recruitment of numerous paraspeckle proteins, including PSF and NONO, to maintain paraspeckle stability, and integrity. We observed improved cell viability and suppressed apoptosis through DNA damage caused by oxidative stress following *NEAT1* knockdown. Thus, our results therefore suggest that *NEAT1* promotes the DNA damage response to cell apoptosis. *NEAT1* is a p53 target gene that plays a role in tumor suppression (Mello et al., 2017). Since the repressive effect of ER on *NEAT1* expression level was suggested by RNA‐seq data, *NEAT1* repression by ER may be involved in the preventive role of ER against oxidative stress (Behl, 2002). Clinically, *NEAT1* is upregulated in AD brain tissues compared to non‐AD tissues. Therefore, these results indicated that *NEAT1* may promote the development of AD. We also investigated the role of NONO in PSF and G3BP2 activity. We observed that the PSF/G3BP2 target genes were not significantly modulated by NONO knockdown. This result suggests that the PSF core action in maintaining neuronal activity is independent of NONO. Therefore, we assume that nuclear effect of PSF/G3BP2 may be independent of paraspeckle.

In summary, our results indicate that PSF and G3BP2 expression are markedly reduced by sporadic AD development and aging. Nuclear interactions between G3BP2 and PSF enhance essential signals for neural activity and maintain cell viability. We propose that decreased function of the RBP complex is important for aging and AD development in the human brain. The gradual loss of fine genetic regulation in aging neuronal cells may explain the neurodegeneration observed in patients with sporadic AD. Thus, the reactivation of the RBP network could be a potential strategy for rescuing cognitive decline in elderly individuals.

## 4 METHODS

### 4.1 Cell culture

U2OS and SH‐SY5Y cells were obtained from the American Type Culture Collection (ATCC). NB1 cells were obtained from Nihon University. U2OS cells were cultured in Dulbecco's modified Eagle medium (DMEM) including 10% fetal bovine serum (FBS), 50 U/mL penicillin, and 50 μg/mL streptomycin. NB1 cells were cultured in Roswell Park Memorial Institute (RPMI) medium including 10% FBS, 50 U/mL penicillin, and 50 μg/mL streptomycin. SH‐SY5Y cells were cultured in advanced DMEM/F‐12 (ThermoFisher) medium including 5 mM L‐glutamine, 10% FBS, 50 U/mL penicillin, and 50 μg/mL streptomycin. All cell lines were cultured at 37°C in a 5% CO_2_ atmosphere. We checked cells for Mycoplasma contamination routinely by Mycoplasma Detection Kit (Jena Bioscience, Jena, Germany). The cell cultures were subcultured at least once monthly using new vials of low‐passage cells stocked for the experiments.

### 4.2 Mice

Three‐ (young), and 24‐months‐old (aged) male or female C57BL/6 J mice were used. All the mice were prepared by the Animal Center of the Tokyo Metropolitan Institute of Geriatrics and Gerontology. All mice used in this study were housed in a specific pathogen‐free (SPF) mouse facility of Tokyo Metropolitan Institute of Geriatrics and Gerontology. The condition for the maintenance was a temperature of 22  ±  2°C, a relative humidity of 55 ± 5%, and a 12:12 h light:dark cycle (lights on, 08:00–20:00). This study was conducted in accordance with ARRIVE guidelines (https://arriveguidelines.org/). The Institutional Animal Care and Use Committee of the Tokyo Metropolitan Institute of Geriatrics and Gerontology inspected and approved our animal experiments (approval no. 2004–2).

### 4.3 Clinical samples

Postmortem brain samples from patients diagnosed with AD or non‐AD controls (non‐AD) were obtained from the Brain Bank of the Tokyo Metropolitan Institute of Geriatrics and Gerontology (https://www.tmig.or.jp/eresearch/a23.html). The protocol for clinical research studies was approved by the Ethics Committees of the Tokyo Metropolitan Institute of Geriatrics and Gerontology (R21‐059) and Tohoku University (2021‐1‐843). Our studies were conducted according to the relevant guidelines and regulations. All the brain tissue samples used in this study were anonymized.

### 4.4 Antibodies

The anti‐NeuN (ab104224), anti‐γH2AX (ab11174), anti‐cleaved PARP1 (ab32064), G3BP1 (ab181150), G3BP2 (ab86135), and anti‐HA (ab9110) antibodies were purchased from Abcam (Cambridge, UK). Mouse monoclonal anti‐β‐actin (A2228) and PSF (WH0006421M2) antibodies were purchased from Sigma‐Aldrich. The mouse monoclonal anti‐Flag (01422383) antibody was obtained from WAKO (Tokyo, Japan). The rabbit polyclonal anti‐PTAU(AT8) (MN1020) antibody was purchased from Thermo Fisher Scientific. The rabbit polyclonal SIRT1 (07131) was purchased from Millipore (Burlington, MA, USA). The rabbit polyclonal PSF (NB100‐61045) was purchased from Novus Biologicals (Centennial, CO, USA). Mouse monoclonal anti‐G3BP1 (611126) and anti‐NONO (611279) antibody was obtained from BD Biosciences (Franklin Lakes, NJ, USA). Mouse monoclonal Amyloid‐β (#10027) was obtained from IBL (Fujioka, Japan). Anti‐phosphorylated‐Tau8 (AT8:90206) was purchased from Fujirebio (Tokyo, Japan).

### 4.5 Transfection and small interfering (siRNA)

Transfection of siRNA was performed using Lipofectamine RNAi MAX (ThermoFisher) as instructed by the manufacturer's protocols. The siRNAs targeting SIRT1 were purchased from Thermo Fisher Scientific (siRNA ID: s223591 (#1) and s223593 (#2)). The siRNAs targeting NEAT1 were purchased from Thermo Fisher Scientific (siRNA ID: n272460 (#1) and n272455 (#2)). The control siRNAs, siPSFs, siNONO, and siG3BP2s have been described previously (Ashikari et al., 2017; Takayama et al., 2017, 2019).

### 4.6 Western blotting

BCA assay kit (Pierce, Tokyo, Japan) was used to determine the protein concentration. The prepared lysates were separated by sodium dodecyl sulfate‐polyacrylamide gel electrophoresis (SDS‐polyacrylamide gels), separated by electrophoresis and transferred electrophoretically. The membranes were reacted with specific primary antibodies at 4°C overnight. We washed membranes with Tris‐buffered saline containing Tween (TBS‐T). Then the membranes were incubated with secondary antibodies for 1 h. Western blotting reagents (Pierce) were used for the detection of antibody–antigen complexes. For IP, lysates were rotated with the indicated antibodies overnight at 4°C. After 2 h of mixing with protein G agarose (GE Healthcare), the beads were washed thrice with lysis buffer, and dissolved into 1× sample buffer (Nacalai). We boiled the mixture at 100°C for 5 min for loading. For nucleus/cytoplasm fractionation, cells were resuspended in 0.5 mL hypotonic solution (10 mM HEPES‐KOH [1 M, pH 7.9], 10 mM KCl, and 1 mM dichlorodiphenyltrichloroethane [DTT]). After incubation for 15 min, we added 1% NP40 and homogenized cells by vortexing. The solution was centrifuged (1000 rpm for 5 min) to pellet the nuclei. The supernatant (cytoplasmic fraction) was recentrifuged (15,000 rpm for 3 min) to remove pellet debris. The nuclear fraction was lysed using RIPA buffer (50 mM Tris–HCl Buffer (pH 7.6), 150 mM NaCl, 1% NP40, 0.5% sodium deoxycholate, and 0.1% SDS).

### 4.7 qRT‐PCR

Total RNA was extracted using ISOGEN II (Nippon Gene), in accordance with the manufacturer's protocol. Prime Script (TAKARA Bio, Kyoto, Japan) was used for yielding complementary DNA from an equal amount of total RNA according to the manufacturer's instructions. PCR studies were performed using a StepOne PCR System (Thermo Fisher) with KAPA SYBR Green (Sigma‐Aldrich). Expression levels of the experimental samples were calculated by the ΔΔCt method relative to the control gene (human *GAPDH* or mouse β‐actin). The primer sequences are listed in Table S1 and have been described previously (Ashikari et al., 2017; Takayama et al., 2017, 2019).

### 4.8 IHC

The formalin‐fixed tissues were embedded in paraffin and sectioned. Tissue sections were deparaffinized, rehydrated in series of graded ethanol, and rinsed with pure water. Antigen retrieval was conducted by incubating the slides in a water bath at 90°C for 30 min in citric acid buffer (2 mM citric acid and 9 mM trisodium citrate dihydrate [pH 6.0]). A Histofine kit (Nichirei), which is based on the streptavidin–biotin amplification method, was purchased for IHC. After blocking the endogenous peroxidase with 3% H_2_O_2_, the sections were reacted with the primary antibodies overnight at 4°C. 3,3′‐diaminobenzidine solution (1 mM 3,3′‐diaminobenzidine, 50 mM Tris–HCl buffer, pH 7.6, and 0.006% H_2_O_2_) was used for visualizing the antigen–antibody complex.

### 4.9 RNA‐seq

We performed RNA‐seq on SH‐SY5Y cells 72 h after treatment with siPSF #2 (10 nM) or siControl to explore the expression profile regulated by siPSF treatment. Total RNA samples with high quality (RNA integrity score >9.0) were used for poly A‐selected sequencing library preparation by the TruSeq RNA Library Preparation Kit v2 (Illumina, San Diego, CA, USA). The libraries were sequenced using a NovaSeq6000 (Illumina) to obtain 50 bp paired‐end reads. Bowtie 2 v. 2.2.6 was used to remove the rRNA sequences. Mapping to the human genome (hg38) was conducted using TopHat 2.1.0. Alignments were generated in a Sequence Alignment Map (SAM) format from the obtained paired‐end reads. For expression analysis, the Human RefSeq mRNA database was used and the number of mapped sequenced tags was calculated. Expression levels of each transcript were normalized to reads per kilobase of exons per million mapped reads (RPKM) to compare among different samples.

### 4.10 RIP assay

Nuclear fractions of the cells were prepared as described above. Nuclei were lysed with RIP buffer (150 mmol/L KCl, 25 mmol/L Tris–HCl, pH 7.4, 5 mmol/L ethylenediaminetetraacetic acid (EDTA), 0.5% NP40, and 0.5 mM DTT). Lysates were reacted with mouse IgG, anti‐G3BP2, or anti‐PSF (5 μg each) by rotating overnight at 4°C with rotation. Protein‐RNA complexes were precipitated using Protein G Sepharose 4 Fast Flow (GE Healthcare), and bead‐associated RNAs were extracted using ISOGEN reagent.

### 4.11 Statistics and reproducibility

Data are shown as mean ± SD. A two‐sided Mann–Whitney *U* test or Student's *t* test was performed to evaluate the statistical significance between the two groups. A one‐way analysis of variance (ANOVA) with Tukey's post‐hoc test was used to compare more than two samples. The statistical tests are described in the figure legends. Statistical significance was set at *p* < 0.05. The qPCR experiments of cell lines were conducted in technical replicates. Biological replicate samples were used for all other analyses. We used GraphPad Prism ver. 6.0 (La Jolla, CA, USA) and Microsoft Excel (Microsoft Corp., Redmond, WA) for the statistical analyses. All experiments were performed at least twice and similar results were observed.

## AUTHOR CONTRIBUTIONS

K.T. designed the study; K.T. and K.S. performed the experiments; T.S. performed immunohistochemical analysis of clinical samples; Y.S. provided human brain tissues; and K.T. and S.I. wrote the manuscript.

## FUNDING INFORMATION

This work was supported by grants from the Japan Society for the Promotion of Science, Japan (21H04829 (S.I.), 20 K07350 (K.T.) and 22H04923 (CoBiA, K.T. and Y.S.)), the Naito Foundation (K.T.), Takeda Science Foundation (K.T. and S.I.), Integrated Research Initiative for Living Well with Dementia (IRIDE) of the Tokyo Metropolitan Institute for Geriatrics and Gerontology (Y.S. and S.I.), AMED (JP21wm0425019 (Y.S.)), Ministry of Health, Labour and Welfare of Japan (JPMH23FC1008 (Y.S.)).

## CONFLICT OF INTEREST STATEMENT

The authors declare no competing financial interests.

## Supporting information


**Data S1:** Supporting Information.

## Data Availability

RNA‐seq data were deposited in the Gene Expression Omnibus (GEO) repository (www.ncbi.nlm.nih.gov/geo; accession number: GSE256401). All other study data are included in the article and/or supplementary figures.
