# Supplementary material for: Cooperative nuclear action of RNA‐binding proteins PSF and G3BP2 to sustain neuronal cell viability is decreased in aging and dementia
Source: Aging Cell. 2024 Aug 18;23(12):e14316. doi: 10.1111/acel.14316 (PMC11634737; doi:10.1111/acel.14316)
Supplement: Supplementary file 1 — Data S1: Supporting Information. [file ACEL-23-e14316-s001.pdf]

1. Supporting Methods

1.1 IF

Cells were plated and cultured on 12-mm circular coverslips (Matsunami-glass, Osaka, Japan) in 24-well plates. The cells were fixed with 4% paraformaldehyde for 10 min at room temperature, and then permeabilized with 0.5% Triton X-100 in phosphate-buffered saline (PBS) for 2 min. After washing with PBS, the cells were blocked with Blocking One (Nacalai, Tokyo, Japan) for 30 min. Cells were incubated with anti-PSF, G3BP1, and G3BP2 antibody in PBS overnight at 4 °C. After washing three times with PBS, the cells or specimens were reacted with anti-mouse IgG conjugated to Alexa Fluor 546 or anti-rabbit IgG conjugated to Alexa Fluor 488 (Life Technologies) in PBS for 1 h. Nuclei were counterstained with 4', 6-diamidino-2-phenylindole (DAPI). Cells or specimens were washed thrice with PBS and coverslips were mounted in glycerol. Cells were subsequently observed and the images were obtained using a confocal laser scanning microscope (FV10i; Olympus, Tokyo, Japan). For mouse tissue specimens, antigen retrieval was conducted by heating the slides in a water bath at 90 °C for 30 min in citric acid buffer (2 mM citric acid and 9 mM trisodium citrate dihydrate [pH 6.0]) after deparaffinization. An M.O.M immunodetection kit (VECTOR, Burlingame, CA, USA) was used in accordance with the manufacturer's instructions.

1.2 Med-IP

Genomic DNA was prepared from mouse and human brain tissue using a DNeasy Blood & Tissue Kit (Qiagen). Methylated DNA was isolated from 1.0 µg of sonicated DNA by a Methylamp Methylated DNA Capture (MeDIP) Kit (Epigentek) according to the manufacturer's protocol. Briefly, sonicated DNA was loaded to the 5-mC antibody-coated wells and incubated for 2 h at room temperature with shaking. After reaction with proteinase K for 60 min at 65 °C, the DNA was eluted and adjusted to a final volume of 100 µL with nuclease-free water. Fold enrichment compared to the input was determined by qPCR using KAPA SYBR Green PCR master mix and the ABI StepOne system (Thermo Fisher Scientific). The human and mouse *GAPDH* promoter regions were used as negative control loci to normalize. The primer sequences are listed in Table S1.

1.3 TUNEL assay

The DEADEND fluorometric TUNEL system (Promega, Madison, WI, USA) was used for detecting cell apoptosis. Slides were stained according to the manufacturer's protocol. DAPI (Nakarai, Tokyo, Japan) was used to counterstain nuclei. TUNEL-positive cells were imaged and photographed using confocal laser scanning microscopy (Fluoview FV10I; OLYMPUS, Tokyo, Japan). We counted the number of cells in five random fields in the three tumors. Data were shown by calculating the mean ± standard deviation (S.D.).

1.4 *in vitro* binding assay

Using HA- or Flag-tagged PSF and G3BP2 expression vectors, the proteins were overexpressed in 293T cells. For proteins purification, Flag (M2) agarose beads (Sigma-Aldrich) and HA agarose beads (Wako) were used. The slurry was then centrifuged at 1500×g for 1 min. The resin pellets were washed three times with 1 mL of NP40 lysis buffer. After centrifugation and removing supernatant, the protein was eluted twice with 100 μL of elution buffer (0.1 mol/L Glycine-HCl (pH2.4)). We added 10 μL 1 M Tris-HCl buffer (pH10.4) to the samples. *In vitro* binding assays were conducted as described (Takayama et al., 2021). Purified proteins were dissolved in binding buffer (20 mM Tris-HCl (pH 7.5), 150 mM NaCl, 0.1% Triton X-100, 1 mM dithiothreitol, and 1x protease Inhibitor Cocktail (Nacalai)) and incubated with anti-HA beads (Wako) at 4 °C for 2 h by rotating. HA-G3BP2 or HA-PSF were then immunoprecipitated, and bound proteins were analyzed by western blotting as described above.

1.5 MTS assay

Cells (1×10^3) were plated in 96-well plates. After treating with siRNAs, cells were cultured for indicated time period. [3-(4,5-dimethylthiazol-2-yl)-5-(3-carboxymethoxyphenyl)-2-(4-sulfophenyl)-2H-tetrazolium, inner salt] (MTS)-based assays were performed to measure cell growth rate using the CellTiter 96 Aqueous Kit (Promega) according to the manufacture's protocol. The MTS assay was conducted in four wells for each group.

2. Supporting References

Takayama K, Kosaka T, Suzuki T, Hongo H, Oya M, Fujimura T, Suzuki Y, Inoue S. Subtype-specific collaborative transcription factor networks are promoted by OCT4 in the progression of prostate cancer. Nat Commun. 2021;12(1):3766. doi: 10.1038/s41467-021-23974-4.

Supporting Figure Legends

Figure S1. PSF and G3BP2 expression level is declined by aging in mouse brain.

(A) Age-dependent expression changes of *Psf* mRNA in mice brain. Quantitative RT-PCR analysis was performed to measure the mRNA expression of *Psf* in mice brain samples (cerebrum, female: N = 9, male: N=11). Mann-Whitney U-test was performed to determine P-value. Data represents mean ± SD.

(B) Age-dependent expression changes of *G3bp2* mRNA in mice brain. Quantitative RT-PCR analysis was performed to measure the mRNA expression of *G3bp2* in mice brain samples (cerebrum, female: N = 9, male: N=11). Mann-Whitney U-test was performed to determine P-value. Data represents mean ± SD.

(C) Locations of primers for Med-IP assay in the CpG islands of *Psf/Sfpq* promoter are indicated (CPG-1, and CPG-2).

(D) Age-dependent expression changes of Psf, G3bp1/2 protein in mice brain (Cerebrum and hippocampus). Western blot in young and old mice is shown (N=3 for female and male). β-actin was used as loading control.

Figure S2. PSF interacts with G3BP2 predominantly in the nucleus of neuronal cells.

(A) Immunostaining of PSF and G3BP2 in NB1 neuronal cells. Cells were treated with 100 μM AS or 1 mM H_2O_2 for 1h. Bar=10μM. Arrows indicate cytoplasmic granules including G3BP2 and PSF signals.

(B) Interaction of G3BP2 and PSF in the nucleus of neuronal cells. NB1 cells were treated with 100 μM AS or 1 mM H2O2 for 1h. Cell lysates from nuclear and cytoplasmic fraction were immunoprecipitated by anti-G3BP2, anti-PSF or nonspecific IgG. Western blot analysis was performed to evaluate protein expression.

(C) *In vitro* binding assay using purified proteins (PSF and G3BP2) tagged with Flag or HA immunoprecipitated by HA-antibody.

(D) G3BP2 interaction with PSF is dependent on C-terminal region. Four HA-tagged deletion mutants of G3BP2 were constructed as indicated. 293T cells were transfected with Flag-PSF and HA-G3BP2 deletion proteins. Immunoprecipitation with anti-HA antibody and subsequent immunoblots with indicated antibodies were performed.

Figure S3. Distinct roles of C-terminal and N-terminal domain of PSF for cellular localization.

(A) Schematic summary of plasmids of PSF deletion mutants (HA-tagged full length, ΔC, ΔCΔR, RBD, ΔN)

(B) Distinct roles of C-terminal and N-terminal domain of PSF for cellular localization. U2OS cells

were transfected with HA-PSF. After 24 h incubation, cells were treated with 100 μM AS for 1h. Immunofluorescence was performed by using anti-G3BP1 and anti-HA antibody and then specimens were observed by confocal microscope. Transfected PSF-deletion mutants were observed in both nucleus (ΔN) and stress granules (ΔC). Bar =10μm.

(C) RNA-binding region of PSF is important for the interaction between PSF and G3BP2. Five HA-tagged deletion mutants of PSF were constructed as indicated. 293T cells were transfected with Flag-G3BP2 and HA-PSF deletion proteins. Immunoprecipitation with anti-HA antibody and subsequent immunoblots with indicated antibodies were performed.

Figure S4. Induction of apoptosis by silencing PSF and G3BP2 in another neuronal cell line.

(A) Silencing of G3BP2 and PSF expression in another neuronal cells. NB1 cells were treated with siG3BP2 #1, #2 (5 nM), siPSF #1, #2 (10 nM), or siControl as indicated. Protein expression level of G3BP2 and PSF were evaluated by western blot analysis. β-actin was used as loading control.

(B) NB1 cells were treated with siG3BP2 #1 (5 nM), siPSF #1 (10 nM), or siControl as indicated. Total RNA was extracted and qRT-PCR was performed to analyze the expression level of *G3BP2* and *PSF* at mRNA level (N=3). Two-sided t-test was performed to determine P-value (vs. siControl). Data represents mean ± SD.

(C) Silencing of G3BP2 and PSF expression induced apoptosis in neuronal cells. NB1 cells were treated with siG3BP2 #1, #2 (5 nM), siPSF #1 (10 nM), or siControl as indicated for 48 h. Protein expression level of cleaved PARP1, G3BP2 and PSF were evaluated by western blot analysis. β-actin was used as loading control.

(D) NB1 cells were treated with siControl, siPSF #2, or siG3BP2 #1 as indicated. Total RNA was extracted and qRT-PCR was performed to analyze the expression level of G3BP2/PSF-target genes at mRNA level (N=3). Two-sided t-test was performed to determine P-value (vs. siControl). Data represents mean ± SD.

(E) NB9 cells were treated with siControl, siPSF #2, or siG3BP2 #1 as indicated. Total RNA was extracted and qRT-PCR was performed to analyze the expression level of G3BP2/PSF-target genes at mRNA level (N=3). Two-sided t-test was performed to determine P-value (vs. siControl). Data represents mean ± SD. *:P < 0.05, **:P < 0.01, ***P < 0.001.

(F) SH-SY5Y cells were treated with siControl, siNONO #1, #2 (10 nM) as indicated. Total RNA was extracted and qRT-PCR was performed to analyze the expression level of G3BP2/PSF-target genes at mRNA level (N=3). Data represents mean ± SD.

Figure S5. *NEAT1* has a promoting role in DNA damage-triggered apoptosis of neuronal cells.

(A) Silencing of *NEAT1* expression in neuronal cells. SH-SY5Y cells were treated with si*NEAT1* #1,

#2 (10 nM), or siControl as indicated. *NEAT1* mRNA expression level was evaluated by qRT-PCR analysis.

(B) Cell proliferation assay of neuronal cells. SH-Y5Y cells were transfected with siControl, si*NEAT1* #1, #2 (10 nM) as indicated. After 72 h incubation, MTS assay was used to quantify the cell growth rate (N=4, biological independent). **P < 0.01. Two-sided t-test was performed. Data are presented as average ± S.D.

(C) Silencing of *NEAT1* expression repressed apoptosis in neuronal cells. SH-SY5Y cells were treated with siNEAT1 #1, #2 (10 nM), or siControl as indicated for 72 h. Then cells were treated with 1 mM H_2O_2 for 1h. Protein expression level of cleaved PARP1 and γ-H2AX were evaluated by western blot analysis. β-actin was used as loading control.

(D) Repressive effect of estrogen on *Neat1* mRNA expression level in mice brain. RNA-seq data of gene expression in mice brain (N=6) was downloaded (GSE144717). RPKM: Reads per million mapped reads.

Figure S6. Enhancement of mRNA post-transcriptional regulation by G3BP2 and PSF.

(A) U2OS cells were transfected with Flag-PSF and HA-G3BP2 as indicated. After 24 h incubation, cells were harvested for immunoprecipitation by anti-APP, anti-Flag or anti-HA antibody. Western blot analysis was performed to evaluate protein expression. β-actin was used as loading control.

(B) U2OS cells were transfected with Flag-PSF and HA-G3BP2 as indicated. After 24 h incubation, cells were harvested for total RNA extraction. qRT-PCR was performed to analyze the expression level of *APP* and *MAPT* at mRNA level (N=). Two-sided t-test was performed to determine P-value (vs. Vec). Data represents mean ± SD.

(C) Stability of G3BP2/PSF-target transcripts in SH-SY5Y and NB1 cells treated with siPSF, siG3BP2 or siControl. Cells were incubated with 2 μg/ml actinomycin D for the indicated times, and target RNA quantities at different time points were evaluated by qRT-PCR (N=3). Two-sided t-test was performed to determine P-value (vs. siControl). Data represents mean ± SD.

(D) Interaction of G3BP2 and PSF protein with pre-mRNA of G3BP2/PSF-target genes. RNA immunoprecipitation (RIP) assay of G3BP2 and PSF was performed in NB1 cells. Pre-mRNA quantities were evaluated by qRT-PCR (N=3). Normal IgG was used as negative control. *GAPDH*: negative control locus. Two-sided t-test was performed to determine P-value (vs. *GAPDH*). Data represents mean ± SD. *:P < 0.05, **:P < 0.01, ***P < 0.001.

Figure S7. Reduced expression of G3BP1/2 in hippocampus of human AD brain tissues compared with controls.

(A) Hippocampus brain tissues from non-AD controls (N=4) were stained with G3BP1/2.

Representative images of G3BP2 IHC including CA1 or parahippocampal gyrus are shown. Bar = 100 μm

(B) Representative images of G3BP1 IHC including CA1 or parahippocampal gyrus are shown. Bar = 100 μm

(C) Hippocampus brain tissues from AD controls (N=6) were stained with G3BP1/2 and PTAU(AT8). Representative images of IHC including CA1 are shown. Bar = 100 μm

(D) Representative images of IHC in parahippocampal gyrus regions are shown. Bar = 100 μm

(E) Expression level of *NONO* in the brain of AD patients (N = 176, female: 88, male:88) or control (non-AD) (N = 187, female: 85, male: 102). A large transcriptome data (GSE15222) was used. Unpaired t-test was performed to obtain P-values. N.S: not significant. Data represents mean ± SD.

Table S1. Sequences of primers for qPCR analysis

Mouse G3bp2
Fw: GTACAGGTCATGGGCTTGCT
Rv: TTGCACAGGTTCAGGAGACG

Mouse Psf
Fw: GTGGGGAATCTACCTGCTGA
Rv: GAACCCTTTGCCTTTGTTGA

Mouse β-actin
Fw: GCAAGTGCTTCTAGGCGGAC
Rv: AAGAAAGGGTGTAAAACGCAGC

5-mC PCR
Mouse Gapdh
Fw: CTGCAGTACTGTGGGGAGGT
Rv: CAAAGGCGGAGTTACCAGAG

Psf CPG-1
Fw: CGCACGGAGGAGAAGATCTC
Rv: ATTACCGCCAAGACGTGAGG
Psf CPG-2
Fw: GTCCAAAAGGCGGCAAGATG
Rv: GAGATCTTCTCCTCCGTGCG

G3BP2
RIP-PCR
1
Fw: CTTCCGAGGCTTCCGAGTTG
Rv: AATGTCCCTTTCCGACCACC
2
Fw: GGTGTAGCGGCAGAGACATT
Rv: AAACCTCAACCCCACCTGTG

GAPDH
RIP-PCR
Fw: TCTCTGCTCCTCCTGTTCGA
Rv: GAGGGAGAGAACAGTGAGCG

NEAT1
RT-PCR
Fw: CCAGTTTTCCGAGAACCAAA
Rv: ATGCTGATCTGCTGCGTATG

PRKN
RIP-PCR
Fw: ATTTAACCCAGGAGAGCCGC
Rv: GTCATTGACAGTTGGCACCG
RT-PCR
Fw: AGGAAAGTCACCTGCGAAGG
Rv: CTTCATGTGCATGCAGCCTC

NRG3
RIP-PCR
Fw: GGAACTCCAAGTACCCAGGC
Rv: GGAGGGCAGAAGGGAAACTC
RT-PCR
Fw: TGGAATTGTCATCGTGGGCA
Rv: TCACAGGATGCCTTTCCACC

SRRM4
RIP-PCR
Fw: GTTCTGGCGAGGAACCTTCA
Rv: AGTGACGGGAAAAGACAGGC
RT-PCR
Fw: CAGGGAACTCCTTCACCACC
Rv: AAGAGGTGGATCGGCTTTGG

SEMA5B
RIP-PCR
Fw: CAGCCCATCTGGTTAGCTCC
Rv: CCTCACCTACCTGGCTTCAA
RT-PCR
Fw: CCTACAGCGAGATTCCCGTC
Rv: TTCATTCTTCGGGGTGCCTC

INHBA
RT-PCR
Fw: CTTGCTGGTGCTCAGGGTAA
Rv: TTCTGGTCCCCACTCTTCCA

MAPT/TAU
RT-PCR
Fw: GAAAGAATCTCCCCTGCAGACCC
Rv: CCTGCTTCTTCAGCTTCCGCT

5-mC PCR

G3BP2

Fw: AACCCAGCTGTTGGTGAGAG

Rv: CGTGTCCCCAAAGCAGTTTG

PSF

Fw: AGGAAACCTCCCCTAGCCTT

Rv: GAGTGTCTATGGGAGCGACG

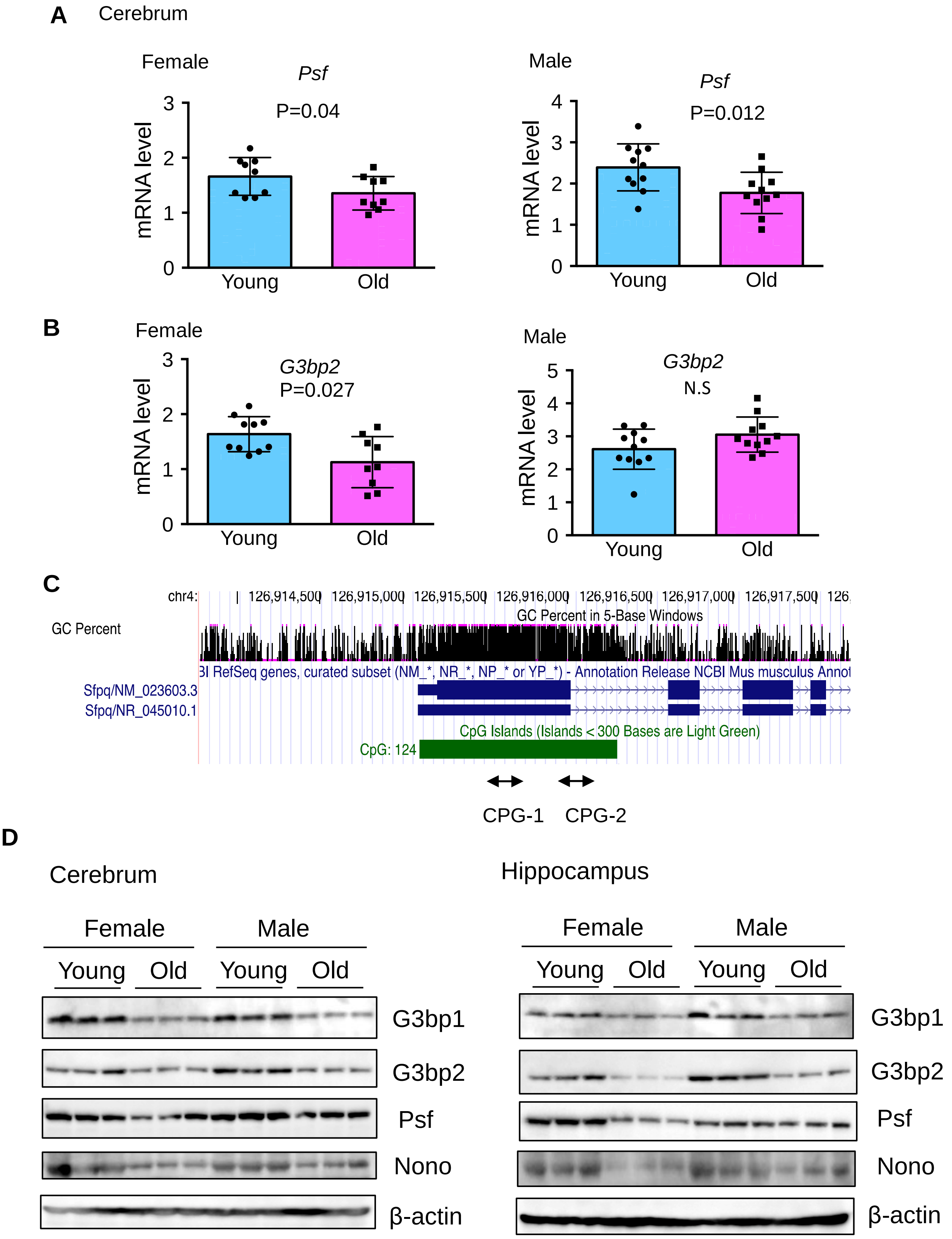
A
Cerebrum
Female
Psf
P=0.04
mRNA level
Young
Old
Male
Psf
P=0.012
B
Female
G3bp2
P=0.027
Male
G3bp2
N.S
C
chr4:
126,914,500
126,915,000
126,915,500
126,916,000
126,916,500
126,917,000
126,917,500
GC Percent
GC Percent in 5-Base Windows
Sfpq/NM_023603.3
Sfpq/NR_045010.1
CpG Islands (Islands < 300 Bases are Light Green)
CpG: 124
CPG-1
CPG-2
D
Cerebrum
Hippocampus
Female
Male
Young
Old
G3bp1
G3bp2
Psf
Nono
β-actin

Figure S1

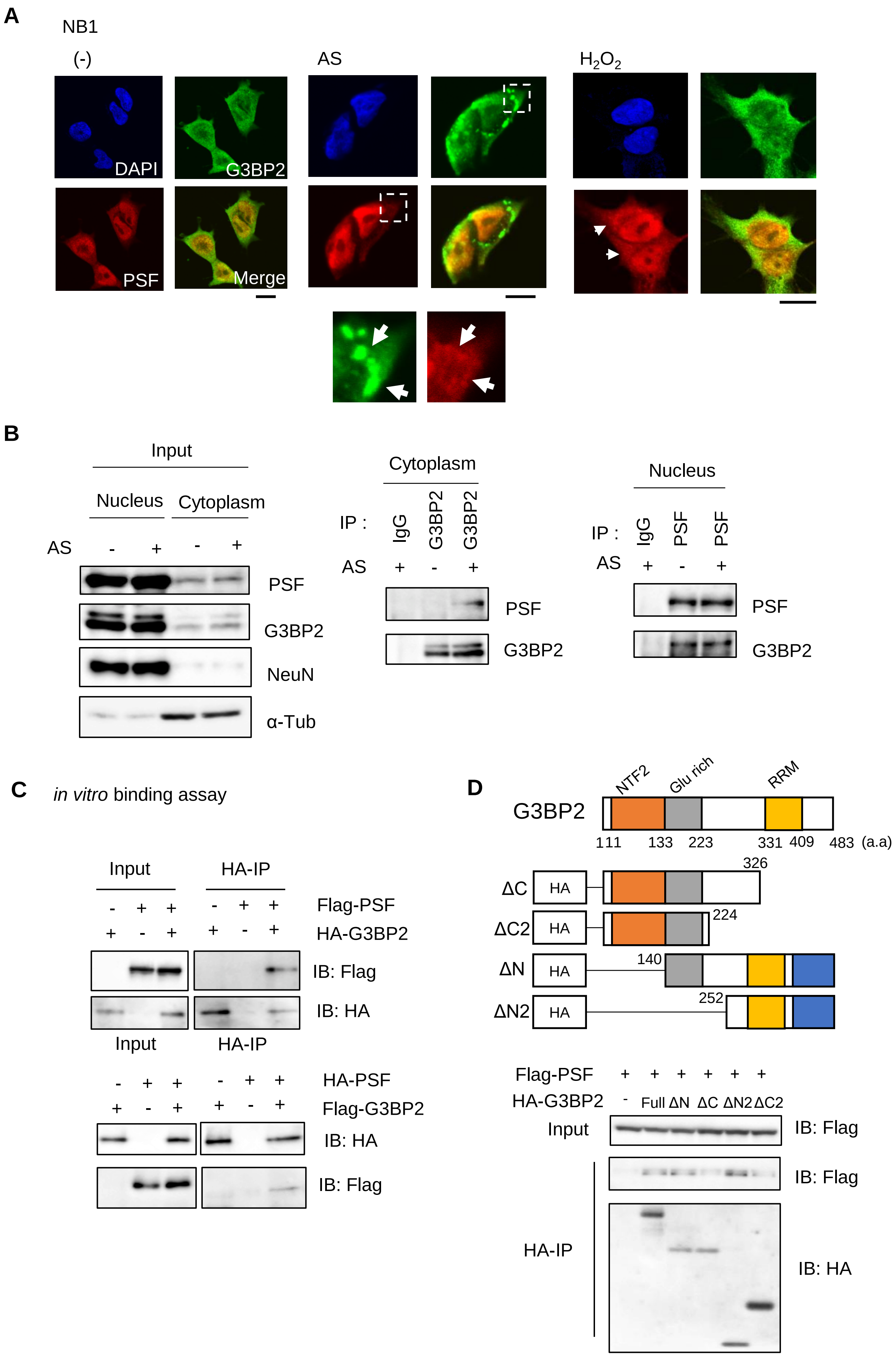
A
NB1
(-)
AS
H2O2
DAPI
G3BP2
PSF
Merge
B
Input
Nucleus
Cytoplasm
AS - + - +
PSF
G3BP2
NeuN
α-Tub
Cytoplasm
IP : IgG G3BP2 G3BP2
AS + - +
PSF
G3BP2
Nucleus
IP : IgG PSF PSF
AS + - +
PSF
G3BP2
C
in vitro binding assay
Input
HA-IP
- + + - + +
Flag-PSF
+ - + + - +
HA-G3BP2
IB: Flag
IB: HA
Input
HA-IP
- + + - + +
HA-PSF
+ - + + - +
Flag-G3BP2
IB: HA
IB: Flag
D
G3BP2
NTF2
Glu rich
RRM
111
133
223
331
409
483
(a.a)
ΔC
HA
326
ΔC2
HA
224
ΔN
HA
140
ΔN2
HA
252
Flag-PSF + + + + + +
HA-G3BP2 - Full ΔN ΔC ΔN2 ΔC2
Input
IB: Flag
IB: Flag
HA-IP
IB: HA

Figure S2

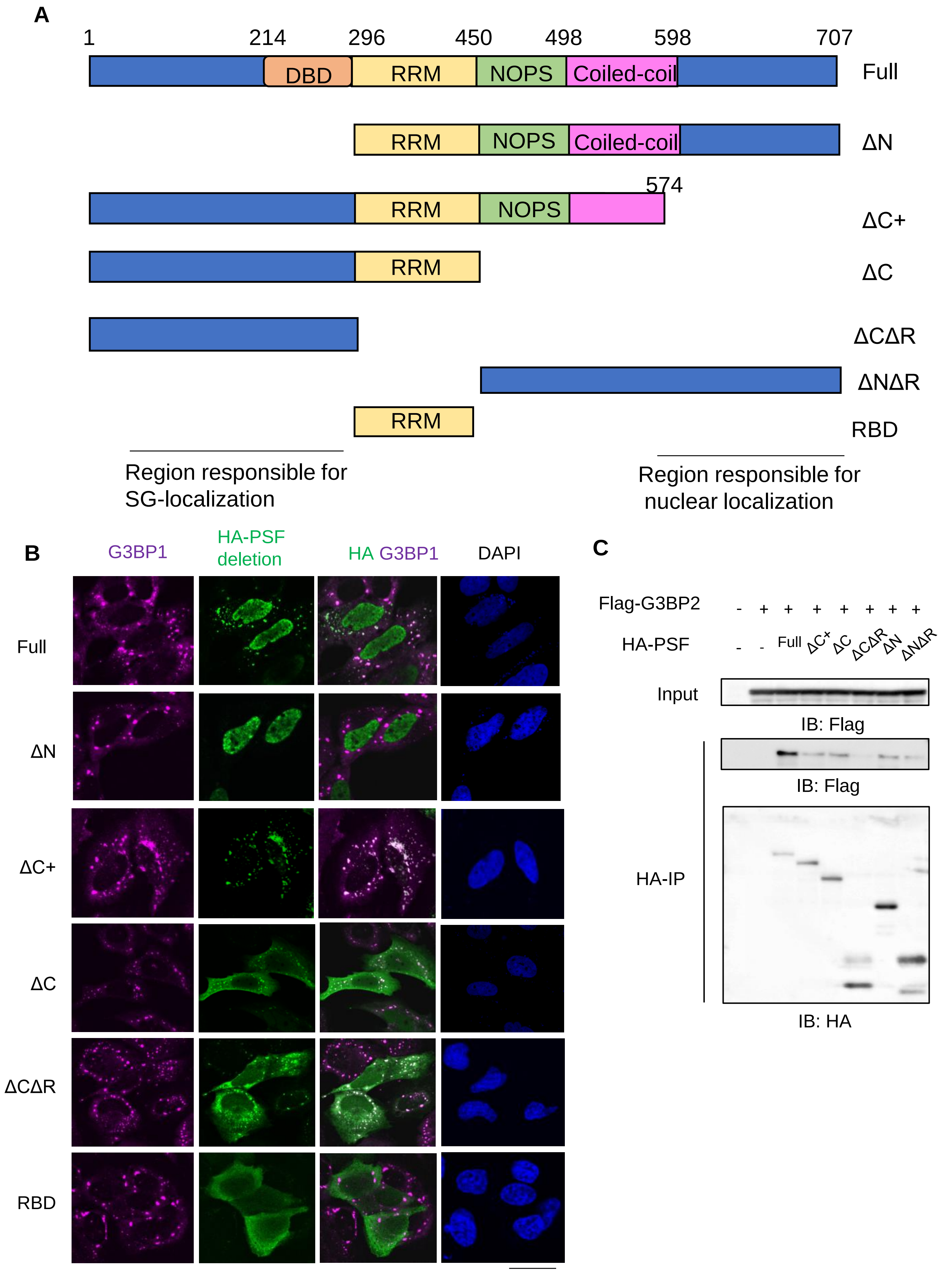
A
1
214
296
450
498
598
707
DBD
RRM
NOPS
Coiled-coil
Full
ΔN
574
ΔC+
ΔC
ΔCΔR
ΔNΔR
RBD
Region responsible for SG-localization
Region responsible for nuclear localization
B
G3BP1
HA-PSF deletion
HA G3BP1
DAPI
Full
ΔN
ΔC+
ΔC
ΔCΔR
RBD
C
Flag-G3BP2
HA-PSF
Full
ΔC+
ΔC
ΔCΔR
ΔN
ΔNΔR
Input
IB: Flag
IB: Flag
HA-IP
IB: HA

Figure S3

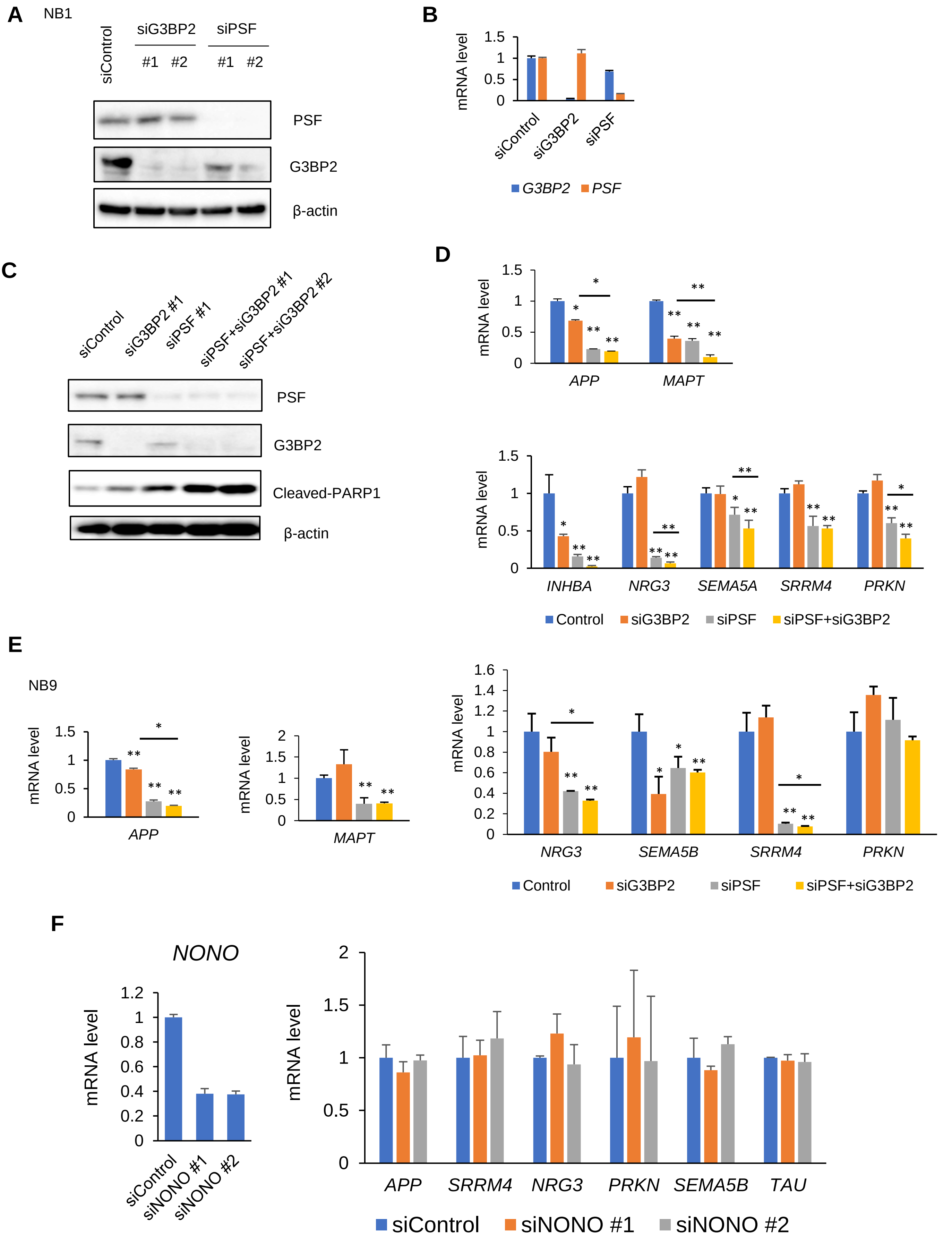
A
NB1
siControl
siG3BP2
siPSF
#1 #2
#1 #2
PSF
G3BP2
β-actin
B
mRNA level
siControl
siG3BP2
siPSF
G3BP2
PSF
C
siControl
siG3BP2 #1
siPSF #1
siPSF+siG3BP2 #1
siPSF+siG3BP2 #2
PSF
G3BP2
Cleaved-PARP1
β-actin
D
mRNA level
APP
MAPT
INHBA
NRG3
SEMA5A
SRRM4
PRKN
Control
siG3BP2
siPSF
siPSF+siG3BP2
E
NB9
mRNA level
APP
MAPT
NRG3
SEMA5B
SRRM4
PRKN
Control
siG3BP2
siPSF
siPSF+siG3BP2
F
NONO
mRNA level
siControl
siNONO #1
siNONO #2
APP
SRRM4
NRG3
PRKN
SEMA5B
TAU
siControl
siNONO #1
siNONO #2

Figure S4

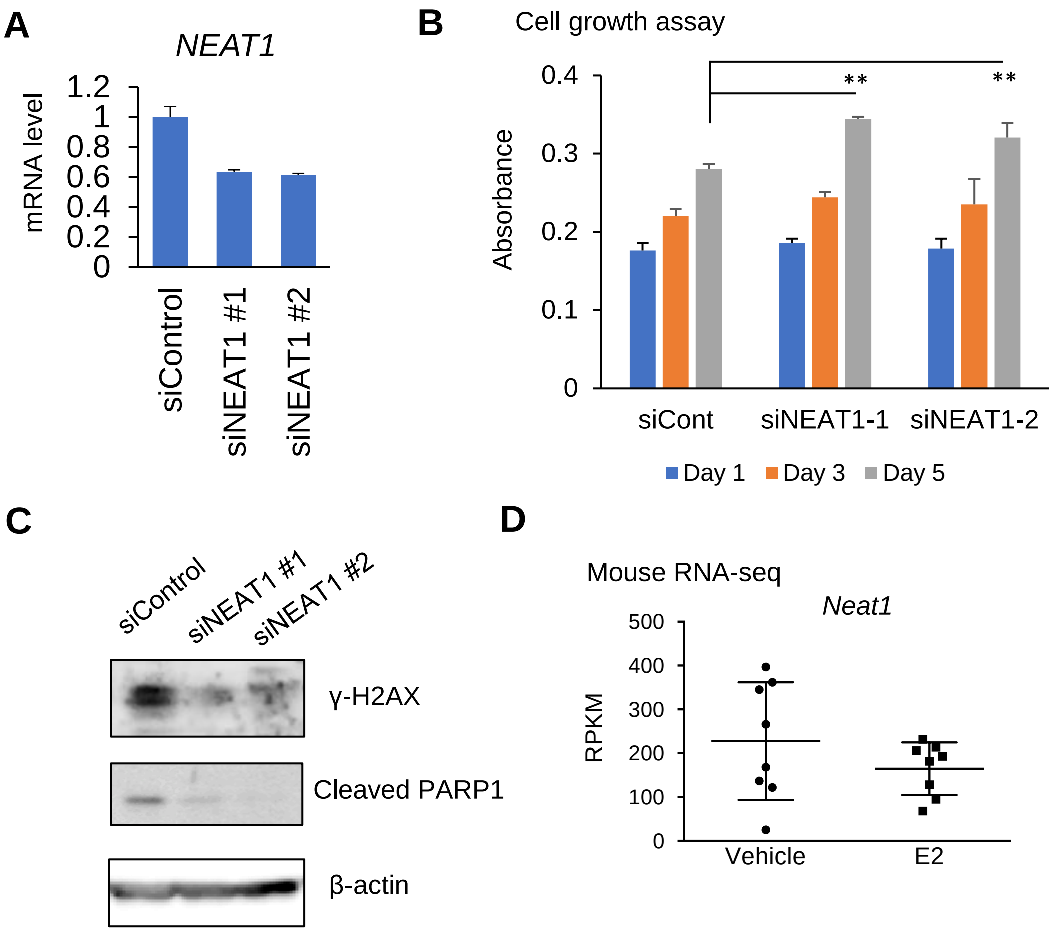
A
NEAT1
mRNA level
1.2
1
0.8
0.6
0.4
0.2
0
siControl
siNEAT1 #1
siNEAT1 #2
B
Cell growth assay
Absorbance
0.4
0.3
0.2
0.1
0
**
**
siCont
siNEAT1-1
siNEAT1-2
Day 1
Day 3
Day 5
C
siControl
siNEAT1 #1
siNEAT1 #2
γ-H2AX
Cleaved PARP1
β-actin
D
Mouse RNA-seq
Neat1
RPKM
500
400
300
200
100
0
Vehicle
E2

Figure S5

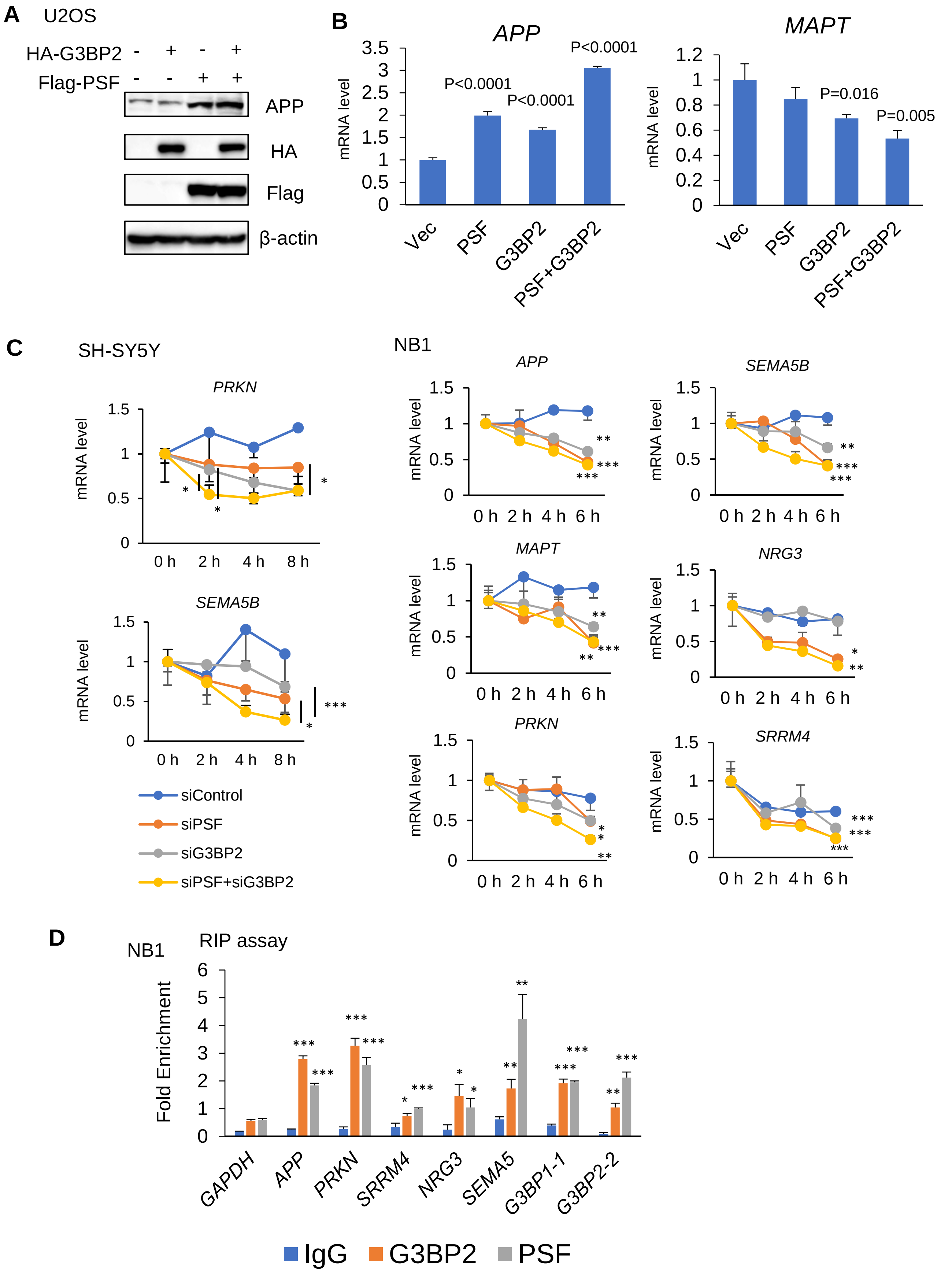

A
U2OS
HA-G3BP2 - + - +
Flag-PSF - - + +
APP
HA
Flag
β-actin
B
APP
MAPT
mRNA level
P<0.0001
P<0.0001
P<0.0001
P=0.016
P=0.005
Vec
PSF
G3BP2
PSF+G3BP2
C
SH-SY5Y
NB1
PRKN
SEMA5B
APP
MAPT
NRG3
SRRM4
mRNA level
0 h 2 h 4 h 8 h
0 h 2 h 4 h 6 h
siControl
siPSF
siG3BP2
siPSF+siG3BP2
D
NB1
RIP assay
Fold Enrichment
GAPDH
APP
PRKN
SRRM4
NRG3
SEMA5
G3BP1-1
G3BP2-2
IgG
G3BP2
PSF

Figure S6

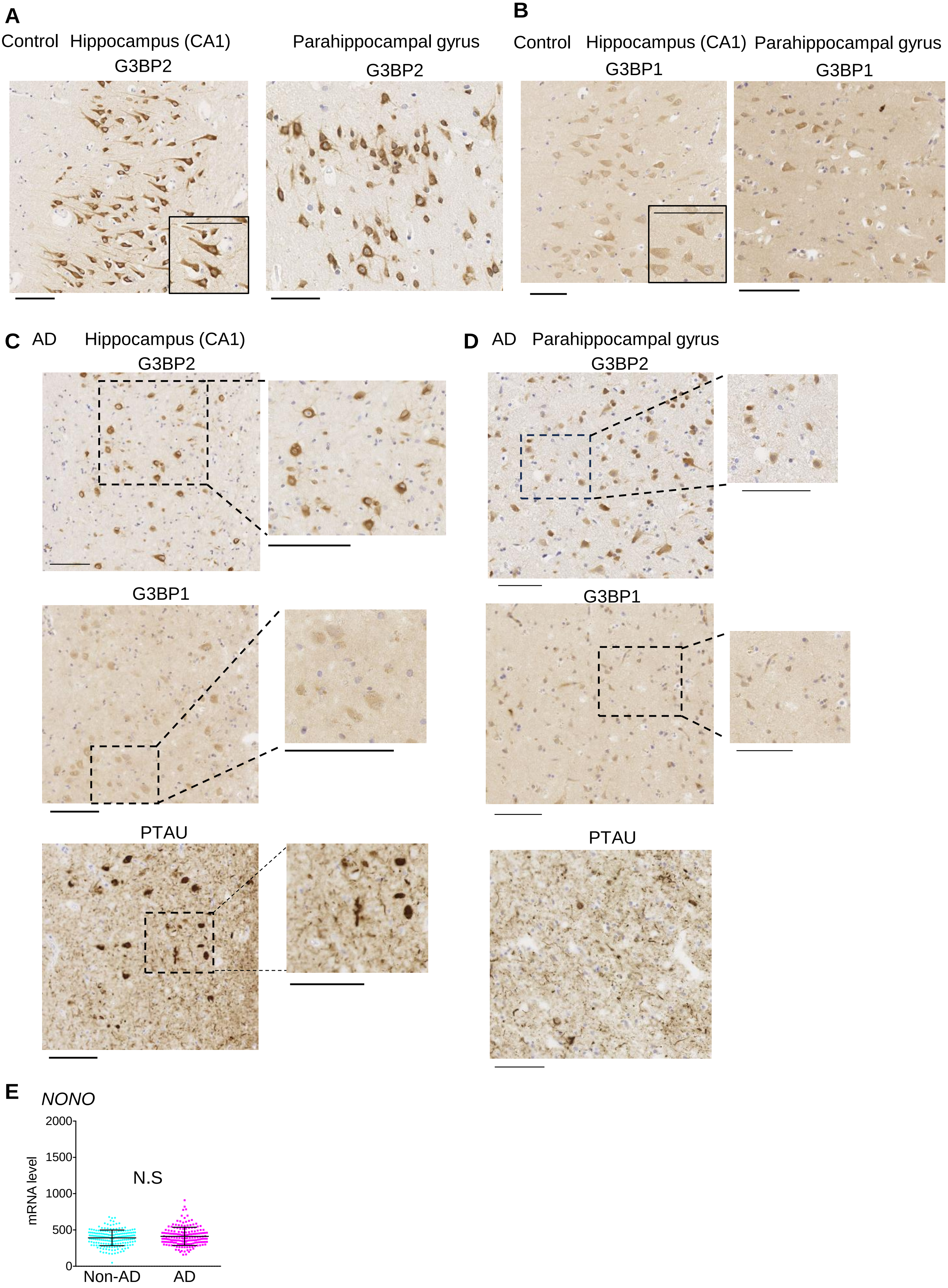
A
Control
Hippocampus (CA1)
G3BP2
Parahippocampal gyrus
G3BP2
B
Control
Hippocampus (CA1)
G3BP1
Parahippocampal gyrus
G3BP1
C
AD
Hippocampus (CA1)
G3BP2
G3BP1
PTAU
D
AD
Parahippocampal gyrus
G3BP2
G3BP1
PTAU
E
NONO
mRNA level
2000
1500
1000
500
0
N.S
Non-AD
AD

Figure S7
